# Tomato salt tolerance mechanisms and their potential applications for fighting salinity: A review

**DOI:** 10.3389/fpls.2022.949541

**Published:** 2022-09-14

**Authors:** Meng Guo, Xin-Sheng Wang, Hui-Dan Guo, Sheng-Yi Bai, Abid Khan, Xiao-Min Wang, Yan-Ming Gao, Jian-She Li

**Affiliations:** ^1^School of Agriculture, Ningxia University, Yinchuan, China; ^2^Key Laboratory of Modern Molecular Breeding for Dominant and Special Crops in Ningxia, Yinchuan, China; ^3^Ningxia Modern Facility Horticulture Engineering Technology Research Center, Yinchuan, China; ^4^Ningxia Facility Horticulture Technology Innovation Center, Ningxia University, Yinchuan, China; ^5^College of Horticulture and Landscape, Henan Institute of Science and Technology, Xinxiang, China; ^6^Department of Horticulture, The University of Haripur, Haripur, Pakistan

**Keywords:** tomato, salinity tolerance, mechanism, abiotic stress, genetic breeding

## Abstract

One of the most significant environmental factors affecting plant growth, development and productivity is salt stress. The damage caused by salt to plants mainly includes ionic, osmotic and secondary stresses, while the plants adapt to salt stress through multiple biochemical and molecular pathways. Tomato (*Solanum lycopersicum* L.) is one of the most widely cultivated vegetable crops and a model dicot plant. It is moderately sensitive to salinity throughout the period of growth and development. Biotechnological efforts to improve tomato salt tolerance hinge on a synthesized understanding of the mechanisms underlying salinity tolerance. This review provides a comprehensive review of major advances on the mechanisms controlling salt tolerance of tomato in terms of sensing and signaling, adaptive responses, and epigenetic regulation. Additionally, we discussed the potential application of these mechanisms in improving salt tolerance of tomato, including genetic engineering, marker-assisted selection, and eco-sustainable approaches.

## Introduction

Salinity has already affected more than one-third of irrigated areas, and it is expected that by 2050, more than half of the world’s cultivated land would be salinized (FAO, 2011; Zhao et al., 2020). Soil salinization severely limits the land use and affects crop yields significantly (van Zelm et al., 2020). Therefore, salt stress has become one of the major abiotic factors threatening food security worldwide. Although the saline-alkali land improvement technology helps in the expansion of arable land, the cost factor severely restricts its application. Cultivating salt-tolerant crops is a worth exploring direction to remediate this thorny problem. A clear understanding of the mechanisms mediating salt tolerance will accelerate the development of new varieties with enhanced salt tolerance.

Salt stress is commonly caused by high concentrations of sodium ion (Na^+^) and chloride ion (Cl^–^) in the soil solution, and its adverse effects on plants include primary stresses like osmotic stress and ion imbalance, as well as secondary stresses like oxidative stress and metabolic abnormalities (Yang and Guo, 2018a). The excessive accumulation of Na^+^ causes the soil’s osmotic pressure to rise, water potential to drop, and root water uptake to decrease, thereby reducing water availability (Julkowska and Testerink, 2015; van Zelm et al., 2020). Furthermore, the Na^+^/potassium ion (K^+^) ratio is disrupted, inhibiting the activities of numerous K^+^-dependent enzymes in the cells (Wu et al., 2018). Chloride harms the plants primarily by causing an ion imbalance via interfering with the uptake or metabolism of other necessary ions (Bazihizina et al., 2019; Zhao et al., 2020). Salt-induced osmotic stress and ion imbalance may not only impair the photosynthesis, thus affecting plant energy metabolism, but also trigger the production of reactive oxygen species (ROS), resulting in oxidative damage.

Tomato (*Solanum lycopersicum* L.), one of the most important vegetable crops, is grown all over the world. Its fruits are widely used as food in the fresh market, and it’s also a model plant for genetics, fruit development, and stress tolerance research (Rothan et al., 2019). Tomatoes are native to western South America, and their wild relatives have been adapted to severely saline coastal regions, however, cultivars have lost their salt resistance during domestication (Gálvez et al., 2012; Pailles et al., 2020). Salt tolerance is a complex trait, thus developing salt-tolerant cultivars necessitates a thorough study of physiological responses, metabolic changes, and gene expression patterns under salinity. To date, the vast majority of salt-tolerance research has been performed using model plants such as *Arabidopsis*, whose contributions to application-oriented research into salt-tolerance are limited by its inherently low levels of salt tolerance and lack of agronomically relevant yield-related traits (Morton et al., 2019). Tomato is a moderately salt-sensitive and yield-targeted crop that can overcome this limitation of *Arabidopsis*. In this review, we provide a critical review of the effects of salt stress, mechanisms of salt tolerance, and biotechniques to improve salt tolerance in tomato. Finally, we highlight research directions for accelerating the genetic improvement of salt tolerance in tomato. Understanding the response and tolerance of tomato to salt stress will enable translational applications to other yield-targeted crops, thus will contribute to the expansion of arable land, agricultural sustainability and global food security.

## Impact of salinity on tomato

### Morphological changes

Generally, the seed germination and early seedling growth stages are most susceptible to salinity, and roots are more vulnerable than other organs (Foolad, 2004). Salt stress delays germination and reduces the germination rate of tomato seeds through changing the activities of key enzymes and the levels of gibberellin (GA), respectively (Singh et al., 2012; Tanveer et al., 2020). Salinity induces the roots to absorb Na^+^, resulting a decrease in the osmotic potential and water intake, which in turn inhibits root growth (Siddiqui et al., 2017; Tanveer et al., 2020). Nevertheless, the root:shoot ratio increased in response to increasing salinity indicating that shoot growth is restricted while root growth is less hindered (Singh et al., 2012). In addition, salinity also represses the development of leaf, flower and fruit by inhibiting cell division or elongation, hindering sugar metabolism and decreasing water import, respectively (Ghanem et al., 2009; Siddiqui et al., 2017; Pinedo-Guerrero et al., 2020).

### Physiological and biochemical changes

Under high salinity stress, tomato plants collect more Na^+^ and Cl^–^, while the levels of K^+^ and calcium ion (Ca^2+^) decrease, disrupting ion homeostasis, which can be restored by rescuing seedlings from salinity (Rahman et al., 2016; Parvin et al., 2019b). Sodium preferentially accumulate in old leaves, protecting young leaves from the toxic effects of saline stress (Khelil et al., 2007). The detrimental effects of Cl^–^ result from interference in the uptake or metabolism of other essential ions, such as nitrate ion (NO_3_^–^) (Zhao et al., 2020).

Excessive ROS induced by high salinity can damage the structure of macromolecules (Waszczak et al., 2018). In roots, oxidative stress caused by salinity was earlier and more sensitive than that in leaves (Gapiñska et al., 2008). The increased anti-oxidase activity in salt-tolerant genotypes helps to protect against oxidative damage (Gharsallah et al., 2016b). Furthermore, the antioxidant defense system and the glyoxalase system work together to detoxify salt-induced ROS and improve salt tolerance in tomato (Parvin et al., 2019b).

Salinity also regulates the compatible solutes, such as proline (Pro) and glycine betaine (GB). Salinity significantly increases Pro, and its level in tomato leaves was higher than that in roots, which attributes in maintaining the chlorophyll content and cell turgidity to protect the photosynthetic activity (Gharsallah et al., 2016b; De la Torre-González et al., 2018). The GB content decreased both in two salt-stressed commercial genotypes, while the levels in the salt-tolerant genotype was lower than that in the sensitive genotype (De la Torre-González et al., 2018). However, exogenous application of GB or its accumulation by genetic engineering alleviated salt-induced K^+^ efflux, enhancing salt tolerance in tomato (Wei et al., 2017).

Short-term salt treatment reduces stomatal conductance, pore area and index, that strongly suppress the dynamic photosynthesis in leaves (Zhang et al., 2018). Under salt conditions, the phytohormones such as abscisic acid (ABA), ethylene (ET), and salicylic acid (SA) are also involved in tomato photosynthesis. ABA causes stomatal closure, reducing leaf gas exchange in salinized plants (Lovelli et al., 2012). ET controls net carbon dioxide (CO_2_) fixation through stomatal or non-stomatal factors, and regulates the activity of photosystems and efficiency of photoprotective processes (Borbély et al., 2020). SA increases photosynthetic activity by inducing higher maximal CO_2_ fixation rate, carboxylation efficiency of Rubisco, and photosynthetic quantum efficiency (Poór et al., 2011).

Unlike the severe damage caused by high salinity, moderate salt stress improves fruit quality by increasing soluble solid content, carotenoid levels, and the accumulation of glutamic acid (Glu), gamma-aminobutyric acid (GABA), glutamine, and α-tocopherol (Massaretto et al., 2018; Meza et al., 2020). The range of salinity to improve fruit quality without affecting tomato yield deserves special attention, which may depend on varieties and duration of stress.

## Mechanisms for salt tolerance in tomato

### Salt signaling pathways and ion transport

How salt enters the plants and salt perception remain unknown (Yang and Guo, 2018b; van Zelm et al., 2020). Ion uptake occurs mainly through the apoplastic and symplastic pathways, and water moves radially through the root via the apoplastic, symplastic and transcellular pathways (the latter two pathways are collectively referred to as cell-to-cell pathway) (Sánchez-Aguayo et al., 2004; Isayenkov and Maathuis, 2019). High salinity in the apoplast alters aqueous and ionic thermodynamic equilibria, which results in ionic and/or osmotic stresses. The percentage of water movement through the symplastic pathway in tomato plants treated with 75 mM NaCl was lower than that in untreated plants. The bulk flow of water and solutes along the apoplastic pathway under saline conditions would impart smaller selectivity and reduced ion uptake (Fernández-García et al., 2002; Sánchez-Aguayo et al., 2004). The contribution of the apoplastic pathway in tomato species that differ in salt tolerance needs to be further explored.

**FIGURE 1:**
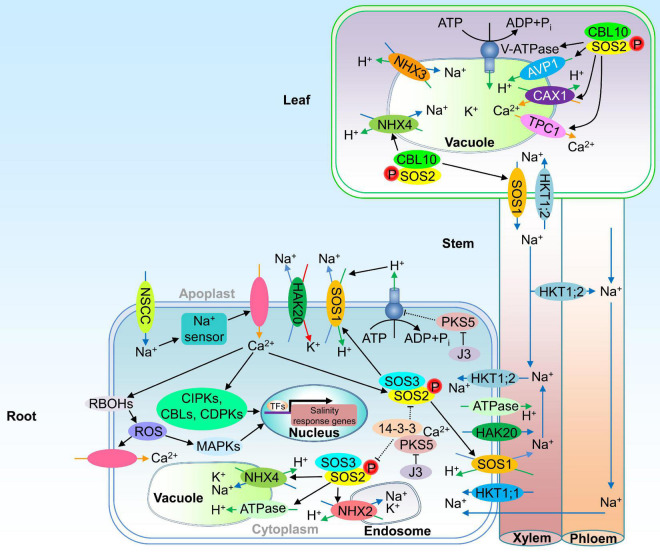
Schematic overview of sodium uptake into tomato roots and transport into leaves. Na^+^ ions enter tomato root cells primarily through NSCC pathway. Na^+^ entering the cell is sensed by a yet undetermined sensory mechanism. Subsequently, Ca^2+^, ROS, and hormone (not shown) signaling pathways are activated. Ca^2+^ induces ROS production by RBOHs and ROS induces Ca^2+^ import. As one part of the Ca^2+^-signaling pathway, CIPKs, CBLs, and CDPKs become active and alter the global transcriptional profile in the nucleus. MAPKs activated by Ca^2+^-ROS- signaling pathway also transduce downstream gene transcription in the nucleus. These early signaling pathways result in activation of detoxification mechanisms. Cytosolic Ca^2+^ ions activate SOS3. SOS2 regulates ATPase, NHX2 and NHX4, increasing Na^+^/H^+^ and K^+^/H^+^ anti-transport activity in root vacuole and endosome. The kinase activity of SOS2 is negatively regulated by 14-3-3 proteins, and the inhibition on SOS2 is released by the Ca^2+^-mediated binding of PKS5 with 14-3-3. SOS1 activated by SOS2-SOS3 heterologous kinase complex is responsible for extruding Na^+^ out the root and partitioning Na^+^ in organs. J3 inhibits PKS5 kinase activity, activating the activity of PM H^+^-ATPases and generating a proton gradient required for Na^+^ transport of SOS1. CBL10-SOS2 complex triggers the separation of Na^+^ into leaf vacuole and the transport of Na^+^ ions from leaves to xylem, activates the tonoplast targets TPC1 and AVP1, maintaining an appropriate Na^+^/Ca^2+^ ratio and V-ATPase, and promotes the proton gradient necessary to energize the Ca^2+^ transport toward the vacuole through CAX1. HKT1;2 is involved in xylem Na^+^ unloading and Na^+^ uploading into the phloem, thus promoting Na^+^ recirculation from shoots to roots, which can be additionally regulated by HKT1;1 in roots. HAK20 transports and regulates the homeostasis of Na^+^ and K^+^. ADP, adenosine diphosphate; ATP, adenosine triphosphate; AVP1, H^+^-pyrophosphatase; CAX1, CATION EXCHANGER 1; CBL, calcineurin B-like protein; CDPKs, calcium-dependent protein kinases; CIPKs, CBL-interacting protein kinases; RBOHs, respiratory burst oxidase homologs; HAK, high-affinity K^+^ transporter; HKT, high K^+^ affinity transporter; J3, DNAJ HOMOLOG3; MAPK, mitogen-activated protein kinase; NHX, Na^+^/H^+^ exchanger; NSCCs, non-selective cation channels; PKS5, PROTEIN KINASE5; ROS, reactive oxygen species; SOS, salt overly sensitive; TFs, transcription factors; TPC1, TWO-PORE CHANNEL 1. The dashed lines indicate that the negative regulatory roles are released under salt stress.

Various transporters are involved in Na^+^ uptake and movement across the plasma membrane (PM), of which non-selective cation channels (NSCCs) are a main route of Na^+^ influx into glycogenic plant roots (Isayenkov and Maathuis, 2019; Figure 1). NSCCs are regulated by different salt-induced early signals, such as Ca^2+^, 3′,5′-cyclic guanosine monophosphate (cGMP), and ROS (van Zelm et al., 2020). K^+^ transporters and transporters from the HKT family are also involved in primary sodium influx into roots (Kronzucker and Britto, 2011). When Na^+^ enters the cell through NSCCs, the membrane depolarizes. This change in membrane voltage prevents the hyperpolarization and tomato *high-affinity K^+^ transporter 5* (*HAK5*) expression induced by K^+^ starvation (Nieves-Cordones et al., 2008). High Ca^2+^ reduces the Na^+^-induced depolarization in tomato root cells and mitigates the repression of HAK5 mediated high-affinity K^+^ uptake (Bacha et al., 2015).

**FIGURE 2:**
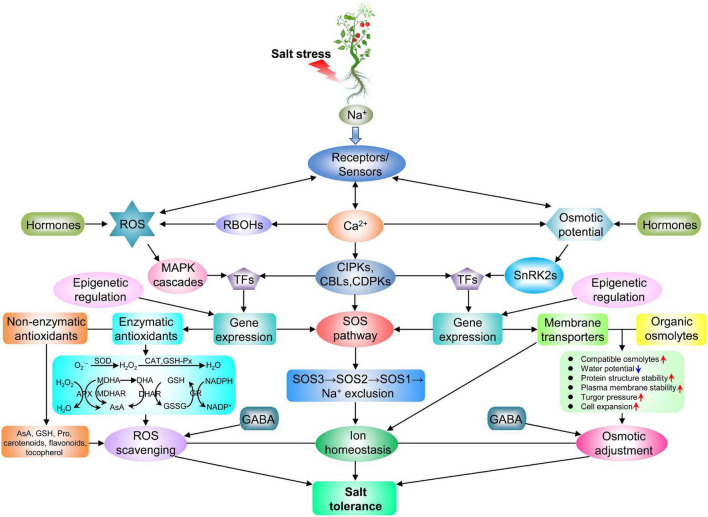
The potential mechanisms underlying salt tolerance in tomato. Salt stress is sensed by unknown receptors or sensors that act synergistically with Ca^2+^ waves, ROS production and osmotic potential. These changes activate antioxidant, ion transporter and osmoregulation pathways, leading to salt tolerance through ROS scavenging, ionic homeostasis and osmotic adaptation. Endogenous GABA participates in ROS scavenging and osmotic adaptation under salt conditions. The red and blue arrows in the box indicate the increase and decrease of the indicators, respectively. APX, ascorbate peroxidase; AsA, ascorbic acid; CAT, catalase; DHA, dehydroascorbate; DHAR, dehydroascorbate reductase; GABA, gamma-aminobutyric acid; GR, glutathione reductase; GSH, glutathione; GSH-Px, glutathione peroxidase; GSSG, oxidized glutathione; H_2_O_2_, hydrogen peroxide; MDHAR, monodehydroascorbate reductase; NADP^+^, oxidized form of NADPH; NADPH, nicotinamide adenine dinucleotide phosphate; O_2_^⋅–^, superoxide radical; Pro, proline; SnRK2s, sucrose non-fermenting-1-related protein kinase 2s; SOD, superoxide dismutase. The remaining abbreviations mentioned in this figure exist in Figure 1.

Plants sense and respond to salt stress within a short period of time. But so far, no specific salt sensor has been identified in cells. The glucuronosyltransferase encoded by *monocation-induced [Ca^2+^]_i_ increases 1* (*MOCA1*) is involved in the biosynthesis of glycosyl-inositol phosphorylceramide (GIPC) sphingolipids at the PM. GIPCs directly bind to Na^+^ and regulate the entry of Ca^2+^ into the cytosol, but the detailed signal transduction process remains unclear (Jiang et al., 2019; Zhao et al., 2020). The decreased pectin crosslinking caused by salt is sensed by a receptor-like kinase (RLK) FERONIA (FER), while the downstream signaling of this receptor is not part of the early signaling response (Feng et al., 2018). The response of plants to salt stress may be the result of sensing and integration of multiple signaling pathways (van Zelm et al., 2020; Figure 2).

High salinity rapidly induces the accumulation of cytosolic Ca^2+^, which can form early signal components of salt sensing relays (Isayenkov and Maathuis, 2019). A series of Ca^2+^-dependent proteins, such as calcineurin B-like proteins (CBLs), calcium-dependent protein kinases (CDPKs) and CBL-interacting protein kinases (CIPKs) are involved in the signal decoding of Ca^2+^ influx into the cytoplasm (Zhao et al., 2020). As the best-characterized CBL- CIPK route, Ca^2+^-dependent salt overly sensitive (SOS) pathway governs ionic homeostasis and salt tolerance, and has been postulated as a molecular switch for salt stress responses (Huertas et al., 2012; Zhu, 2016). SOS3, a functional Ca^2+^-binding protein, senses Ca^2+^ and physically interacts with SOS2 at the PM. SOS2 is a serine/threonine protein kinase functioning as a key regulator of ion transporters (Belver et al., 2012). SOS3–SOS2 complex phosphorylates and activates SOS1, a PM Na^+^/H^+^ antiporter that extrudes Na^+^ out of the cell (Yang and Guo, 2018b). Tomato SOS3 is mainly expressed in roots (Cho et al., 2021), and SOS3–SOS2 complex can activate Na^+^/H^+^ antiport activity of *Arabidopsis* AtSOS1 (Huertas et al., 2012), indicating that the SOS pathway is conservative.

In tomato, decreased levels of *SOS1* lead to a salt-sensitive phenotype (Olías et al., 2009a; Wang Z. et al., 2021). Unlike only the overexpression of activated forms of *Arabidopsis AtSOS2* increases salinity tolerance in transgenic plants (Guo et al., 2004), overexpression of the both native and activated forms of tomato *SOS2* increase salt tolerance (Huertas et al., 2012; Cho et al., 2021). Activation of SOS1 in tomato plants overexpressing *SOS2* may contribute to the efflux of Na^+^ out the root epidermal cells as well as the active loading of Na^+^ into shoots (Olías et al., 2009a; Belver et al., 2012; Huertas et al., 2012). Overexpression of tomato *enhancer of SOS3-1* (*ENH1*) excludes more Na^+^ from cytosol and transports into vacuoles, and maintains more K^+^ in cytosol to reestablish ion homeostasis (Li D. et al., 2013).

Tomato SOS2 regulates the activity of V-ATPase, energizing the Na^+^/H^+^ antiport at the endosomal-prevacuolar as well as vacuolar compartments. Additionally, SOS2 activates the Na^+^/H^+^ and K^+^/H^+^ antiport activity in root intracellular membranes and the Na^+^/H^+^ antiport activity in root tonoplast vesicles, which are regulated by the endosomal-vacuolar K^+^, Na^+^/H^+^ (NHX2 and NHX4) antiporters (Huertas et al., 2012). The K^+^/H^+^ antiporter NHX2 increases salt tolerance by improving K^+^ uptake and compartmentalization (Rodríguez-Rosales et al., 2008; Huertas et al., 2013). Tomato plants overexpressing both *NHX2* and *SOS2* grow better under salinity conditions than plants overexpressing only one of them (Baghour et al., 2019). How SOS2 activates NHX transporters in tomato roots remains to be clarified.

Under salt conditions, tomato CBL10 can form a complex with SOS2. SOS2–CBL10 complex maintains a proper Na^+^/Ca^2+^ ratio in the vacuole of leaf cells through the activation of tonoplast targets, therefore protecting young developing tissues from the damage caused by salt (Figure 1). The tonoplast targets include the cation channel TWO-PORE CHANNEL 1 (TPC1, mediating Ca^2+^ release from vacuoles) and two vacuolar H^+^-pumps, H^+^-pyrophosphatase AVP1 (AVP1) and V-ATPase (V-ATPase). The expression of tomato *SOS2* is associated with *CBL10*. Loss function of *CBL10* strongly inhibits the expression of *CATION EXCHANGER 1* (*CAX1*) and impairs the proton gradient necessary to energize the Ca^2+^ transport toward the vacuole through CAX1 antiporter (Egea et al., 2018). The role of tomato SOS2-CBL10 complex in regulating vacuolar Na^+^ sequestration is not well characterized.

Plant 14-3-3 proteins are phosphoserine-binding proteins that regulate the activities of a wide array of targets, playing an important role in response to salt stress (Xu and Shi, 2006). 14-3-3 proteins inhibit the kinase activity of SOS2 under normal conditions. PROTEIN KINASE5 (PKS5) inhibits SOS2 activity by promoting the interaction between SOS2 and 14-3-3 proteins. Under salt conditions, chaperone DNAJ HOMOLOG3 (J3) interacts with PKS5 kinase and inhibits its activity, thereby releasing the inhibition of SOS2 activity by 14-3-3 protein, which is associated with salt-induced Ca^2+^ signal (Yang and Guo, 2018b; Yang et al., 2019; Zhao et al., 2020). At least 12 genes named *TOMATO 14-3-3 PROTEIN1 (TFT1)-TFT12* are predicted to encode tomato 14-3-3 proteins, and the levels of *TFT1*, *TFT4*, *TFT7*, and *TFT10* are significant up-regulated by salt (Xu and Shi, 2006). Enhanced salt tolerance in *TFT7*-overexpressing transgenic plants due to the reduction of oxidative stress injury rather than the maintenance and reestablishment of cellular ion homeostasis (Xu and Shi, 2007). In addition to salt stress, tomato *TFT4* is also involved in response to alkali stress, but the functions of tomato *TFT4* in the integration of H^+^ efflux, the basipetal indole-3-acetic acid (IAA) transport, and the PKS5-J3 pathway need to be further defined (Xu et al., 2013).

Salt stress induces the accumulation of ROS, including superoxide radical (O_2_^⋅–^), hydroxyl radical (OH^⋅^), singlet oxygen (^1^O_2_), and hydrogen peroxide (H_2_O_2_) (Yang and Guo, 2018a). ROS have toxic effects at high concentrations, while function as signal transduction molecules at low concentrations (Miller et al., 2010). Under salt conditions, increased cytosolic Ca^2+^ induces respiratory burst oxidase homologs (RBOHs) to generate ROS, and ROS induces Ca^2+^ entry into stomata (van Zelm et al., 2020). In tomato, most of eight *RBOH* genes were significantly upregulated by persistent salt stress, showing that RBOHs are essential regulators response to long-term salinity stress (Raziq et al., 2022). Tomato *RBOH1*-dependent apoplastic H_2_O_2_ mediates epigallocatechin-3-gallate-induced abiotic stress tolerance (Li et al., 2019b), high atmospheric CO_2_-dependent salt stress tolerance (Yi et al., 2015) and spermine-induced salinity-alkalinity stress tolerance (Xu et al., 2021). Furthermore, salt-induced ROS can activate downstream mitogen-activated protein kinase (MAPK) cascades. Activated MPKs transduce signals to downstream transcription factors (TFs) in the nucleus to induce the expression of stress-responsive genes (Isayenkov and Maathuis, 2019). MAPK cascade consists of three consecutively acting and phosphorylating protein kinases, MAP kinase kinase kinase (MAPKKK/MEKK), MAP kinase kinase (MAPKK/MKK), and MAP kinase (MAPK/MPK) (Mishra et al., 2006). In tomato genome, 16 *MAPK*, 5 *MAPKK* and 89 *MAPKKK* genes are identified (Kong et al., 2012; Wu et al., 2014). Tomato *MAPKK2* and *MAPKK5* were significantly upregulated by salt stress, and the transcript levels of 13 *MAPKKK* genes shown a more than 10-fold change under salt stress (Wu et al., 2014). Although there is no evidence that salt stress affects the expression levels of tomato MAPK family genes, *MAPK3* improves salt tolerance by increasing the expression levels of ET synthesis genes and SOS pathway genes (Shu et al., 2022).

Membrane transporters play critical roles in maintaining Na^+^/K^+^ homeostasis (Figure 1). The Na^+^ transporter *HKT1;2* gene is responsible for the major quantitative trait loci (QTL) involved in Na^+^ and K^+^ homeostasis in tomato. Silencing of *HKT1;2* alters the Na^+^/K^+^ ratio, suppresses the response of *SOS1* to salt, and increases salt hypersensitivity (Asins et al., 2013; Jaime-Pérez et al., 2017; Romero-Aranda et al., 2020). HKT1;2 of *S. cheesmaniae* is not only involved in xylem Na^+^ unloading but also involved in Na^+^ uploading into the phloem, thus promoting Na^+^ recirculation from aerial parts to the roots, which can be additionally favored by *HKT1;1* silencing at the roots (Romero-Aranda et al., 2021). Decreasing Na^+^ content in young leaves is probably linked to the up-regulation of *SOS1* contributing to Na^+^ exclusion from the cytosol toward the leaf apoplast. Nevertheless, preventing Na^+^ accumulation in the cytosol of mature/old leaves may be mainly attributed to the combined action of HKT1-like transporters (mediating Na^+^ unloading from the xylem) and NHX-type transporters (promoting Na^+^ and K^+^ accumulation in vacuoles and endosomes) (Olías et al., 2009b; Belver et al., 2012). CBL10 regulates the expression of *HKT1;2* and *SOS1*. The lack of *CBL10* severely inhibits Na^+^ compartmentalization into vacuoles as well as Na^+^ upload from xylem into cells, while promotes Na^+^ extrusion from leaf cells to xylem, dilapidating its functional role in regulating Na^+^ homeostasis and protecting shoot apex and developing tissues from salt damage (Egea et al., 2018). The HAK/KUP/KT (high-affinity K^+^/K^+^ uptake/K^+^ transporter) family transporters primarily mediate K^+^ fluxes. Tomato HAK20 is identified as the major player controlling root Na^+^/K^+^ ratio with genome-wide association studies (GWAS) (Wang et al., 2020). It functions in the loading of K^+^ and Na^+^ into the xylem in roots. The wild allele of *HAK20* loads more Na^+^ into root xylem and enhances Na^+^ efflux, therefore lowering the Na^+^/K^+^ ratio in roots and leading to higher salt tolerance, but its function in shoot is still elusive (Wang et al., 2020; Xiang and Jiménez-Gómez, 2020).

### Osmotic adjustment

High salinity causes ionic imbalance and water deficit in plant cells, leading to osmotic stress (Zhao et al., 2021). A hyperosmolality-gated calcium-permeable channel encoded by *reduced hyperosmolality-induced [Ca^2+^]_i_ increase1 (OSCA1)* was identified as an osmotic stress sensor (Yuan et al., 2014), but the role of OSCA1 in the sense of high salinity-triggered osmotic stress is questioned (Zhao et al., 2020). Plants perform osmotic adjustment by increasing the concentration of solutes and decreasing water potential, which is vital for the alleviation of the osmotic imbalances and for maintaining cell turgor (Lv et al., 2019). Under osmotic stress, solutes can act as osmolytes or play a protective role by stabilizing the structure of biological macromolecules (Figure 2). Osmotic regulators mainly include inorganic ions and organic substances (Hao et al., 2021). The organic osmolytes includes sugar, complex sugars, Pro, GB, polyamines (PAs), polyols and late embryogenesis abundant (LEA) proteins (Yang and Guo, 2018b; Liu et al., 2022). Halophytes and salt-tolerant non-halophyte species synthesize organic solutes for osmotic adjustment in the cytoplasm only, while absorb Na^+^ and Cl^–^ from the soil for the bulk of osmotic adjustment in the vacuole, a more energy-efficient strategy (Munns et al., 2016). Due to low tissue tolerance, a large portion of osmotic adjustment in salt-sensitive species occurs with organic solutes, but the high synthetic cost decreases the growth rate (Munns and Gilliham, 2015).

K^+^ is one of the macronutrients required by plants for growth, and plays an important role in preventing cell damage caused by salt. Exogenous K^+^ activates carbohydrate metabolism and Pro accumulation through endogenous hydrogen sulfide (H_2_S) signaling, thereby increasing osmotic tolerance and enhancing the hydration levels of the salt stressed tomato seedlings (Khan et al., 2021). Under salt conditions, K^+^, Na^+^, Cl^–^, and organic acids are the main osmolytes in tomato plants (Wang et al., 2011). The hydration ability of Pro helps the attached proteins bind more water, and prevents protein dehydration and denaturation under salt stress (Hao et al., 2021). The Pro increase in the tolerant tomato varieties supports the opinion that Pro counteracts the osmotic stress caused by salinity (De la Torre-González et al., 2018). On the other hand, the increased Pro in plants with salt treatment is generally not enough for beneficial effects, and the accumulation of Pro increases as the more stress effects. Thus, Pro concentration can be used as a negative indicator of tolerance. Foliar application of a mixture of Pro and Glu mitigated the negative effects of salt on tomato growth by accumulating total soluble sugars, but the concentration of Pro was significantly decreased (Alfosea-Simón et al., 2020). The beneficial effects of GB on reducing osmotic imbalance induced by salt stress have been demonstrated in various plants (Gupta and Huang, 2014; Lv et al., 2019). However, in some tomato genotypes, GB concentration is negatively correlated with salt tolerance, which may be due to the oxidative stress caused by H_2_O_2_ generated from the synthesis of GB via the choline pathway (De la Torre-González et al., 2018). The roles of Pro and GB in salt tolerance may depend on the species and cultivars. Foliar application of L-methionine (Met) and L-phenylalanine (Phe) induces salt tolerance of tomato by enhancing the PM stability, the contents of osmolytes, and the activity of antioxidative enzymes (Almas et al., 2021). Under salt stress, exogenous GABA inducing amino acid content increases osmotic adjustment capacity to resist water loss and neutralizes excessive Na^+^ in the vacuoles in tomato leaves (Wu et al., 2020). Trehalose alleviates the salt damage of tomato plants by promoting the accumulation of osmotic substances Pro, GB, and soluble proteins (Yang Y. et al., 2022).

Cell wall-associated protein kinases (WAKs) localized in PM induce the changes of solutes contents. Mutation of tomato *WAK1* is tolerant to Na^+^ homeostasis but not to osmotic homeostasis, and it increases sucrose content in roots. The salt sensitivity of *wak1* mutant is due to the altered osmotic and metabolic homeostasis (Meco et al., 2020). Under osmotic stress, the tomato cryptochrome 1a (cry1a) enhances the growth by reducing the MDA content and Pro accumulation, and specific blue light fluence rates are required for cry1a-mediated osmotic responses (D’Amico-Damião et al., 2021). The dehydrin *tas14* gene, a member of the tomato LEA family, improves osmotic tolerance by reducing osmotic potential and accumulating solutes (such as sugars and K^+^). Plants overexpressed *tas14* transfer Na^+^ into adult leaves while K^+^ and sugars in young leaves, achieving osmotic balance in older leaves at a minimal energy cost (Muñoz-Mayor et al., 2012). Overexpression of tomato *ICE1a*, a MYC-type ICE1-like TF, increases the levels of Pro, soluble sugars and LEA proteins, enhancing osmotic and salt tolerance (Feng et al., 2013). In tomato plants, the accumulation of several osmotic protectants (Pro, sucrose, glucose, and GB) under salt conditions, depends on the regulation of key enzymes in their synthetic pathway at both transcriptional and post-transcriptional levels (Rivero et al., 2014).

Endogenous ABA improves short-term osmotic stress resistance in tomato via osmotic and hydraulic adjustments (Li et al., 2022). Tomato ABSCISIC ACID STRESS RIPENING1 (ASR1) protects yeast from osmotic stress by inducing downstream components of the high-osmolarity glycerol pathway (Moretti et al., 2006). ASR1 also binds directly to a tomato cellulose synthase-like (CSL) protein gene that may play key roles in osmotic stress (Ricardi et al., 2014). All the tomato *sucrose non-fermenting 1-related protein kinase 2* (*SnRK2*) genes are salt stress responsive and most of them are also induced by ABA (Yang et al., 2015; Chen et al., 2016). Overexpression of tomato *SnRK2.1* and *SnRK2.2* regulates the expression of stress-related genes and decreases osmotic tolerance, but the regulatory relationship between the activated SnRK2s and stress-related gene expression remains to be identified (Yang et al., 2015). In addition to ABA, ET and auxin also play important roles in osmotic stress response. Under osmotic stress, the ET-induced H_2_S is required for ET-induced tomato stomatal closure (Jia et al., 2018). Overexpression of tomato *ET responsive factor 1* (*TERF1*) in tobacco induces not only the typical ET triple response but also salt tolerance by stimulating the expression of downstream genes. *TERF1* may be a linker in ET and osmotic signaling pathways (Huang et al., 2004). Down-regulation of tomato *Auxin Response Factor 4* (*ARF4*) improves salt and osmotic tolerance by reducing stomatal conductance along with increased leaf relative water content and ABA content (Bouzroud et al., 2020).

### Reactive oxygen species generation and antioxidant defense

#### Reactive oxygen species accumulation

Salt stress rapidly induces the production of ROS in plant apoplast, chloroplasts, mitochondria, and peroxisomes (Chen et al., 2021). The apoplastic ROS are produced by the activation of PM-localized nicotinamide adenine dinucleotide phosphate (NADPH) oxidases (RBOHs), apoplastic peroxidases (PODs), diamine oxidases (DAOs), and PA oxidases (PAOs) (Qi et al., 2017). Salt induces the transcript of tomato *RBOH1* and enhances the activity of NADPH oxidase, deriving H_2_O_2_ accumulation in the apoplast. Inhibition of *RBOH1* expression impairs the ROS scavenging induced by *RBOH1*-dependent H_2_O_2_ signal, while the downstream molecular players that transduce ROS signaling to the transcriptional level remain elusive (Li et al., 2019b). Tomato MAPK3 may increase salt tolerance and decrease heat tolerance through the RBOH1-dependent antioxidant system, respectively (Yu et al., 2019; Shu et al., 2022). How tomato MAPK3 and RBOH1 function in the combined response to salt and heat stress remains to be elucidated. Salt stress improves the activities of DAO and PAO. The terminal oxidation of PAs by DAO and PAO contributes to the production of H_2_O_2_. PAO inhibitor reduced the activity of PAOs and inhibited H_2_O_2_ production, but did not increase salt tolerance due to the significantly increased electrolytic leakage (Takács et al., 2017). PAO induced by spermidine (Spd) mediates the elevation of H_2_O_2_ level, thereby activating the antioxidant system to eliminate excess ROS accumulation and relieve membrane lipid peroxidative damage and growth inhibition under saline-alkali stress (Yang J. et al., 2022). These reports suggest that ROS are primarily used for stress-sensing and signaling (Mittler et al., 2022).

Both salt stress-induced stomatal closure and accumulation of high levels of Na^+^ in the cytoplasm impair the photosynthetic machinery. As a result, the absorbed light exceeds the demand for photosynthesis, leading to the formation of ROS in chloroplasts, including O_2_^⋅–^, ^1^O_2_, and H_2_O_2_. O_2_^⋅–^ is generated by the Mehler reaction in the photosystem I (PSI), ^1^O_2_ is produced by photosystem II (PSII) in the thylakoid membrane because of limitation of the electron transport between photosystems, and H_2_O_2_ is produced at the electron-donor side of PSII via the incomplete oxidation of water (Zhao et al., 2020). In tomato, salt stress induced the increased ROS levels and the decreased PSII activity by reducing the oxygen-evolving complex (OEC) activity on the donor side of PSII, damaging the donor and acceptor sides of the photosystem, and blocking the electron transfer on receptor side of PSII. Melatonin (MT) reduces the production of ROS by balancing the distribution of photosynthetic electron flux and enhances the scavenging ability of ROS by promoting the activities of enzymes involved in the ascorbate glutathione (AsA-GSH) cycle, increasing salt tolerance in tomato (Yin et al., 2019).

Another pathway of ROS production is mitochondrial respiration. Electrons leak from complexes I and III of the mitochondrial electron transport chain to molecular oxygen, resulting in O_2_^⋅–^ generation and then be rapidly catalyzed into H_2_O_2_ (Liu et al., 2020). Peroxisomes are one of the major sites where plants produce intracellular H_2_O_2_ (Liu et al., 2021). SA induces mitochondrial ROS production by reducing mitochondrial hexokinase (mtHXK) activity in tomato leaves (Poór et al., 2019). Salt induces the increase of mitochondrial H_2_O_2_ in the cultivated tomato roots, attributing to the non-enzymatic reduction of superoxide by AsA and GSH, while the decreased H_2_O_2_ of wild salt-tolerant related species is due to the higher rate of H_2_O_2_ detoxification. The decreased H_2_O_2_ in peroxisomes is in part the result of the activities of ascorbate peroxidase (APX) and catalase (CAT) over that of superoxide dismutase (SOD) (Mittova et al., 2004). The gene encoding peroxisome-localized PAO4 is regulated by salt and oxidative stress, suggesting that tomato PAOs may also be involved in ROS metabolism in peroxisomes under salt conditions (Hao et al., 2018).

#### Enzymatic scavenging system

Salt-induced overaccumulation of ROS imposes oxidative stress on plants, causing ionic imbalance, DNA mutation, peroxidation of lipids and carbohydrates, protein denaturation, pigment breakdown and impaired enzymatic activity (Liu et al., 2020). Tomato plants have formed the antioxidant defense system to mitigate ROS stress caused by salt (Figure 2). The antioxidant defense system consists of enzymatic and non-enzymatic antioxidants (Hao et al., 2021). Enzymatic antioxidants include RBOHs, SOD, APX, CAT, glutathione reductase (GR), glutathione S-transferase (GST), glutathione peroxidase (GSH-Px), monodehydroascorbate reductase (MDHAR), dehydroascorbate reductase (DHAR), and guaiacol peroxidase (GPOX). Non-enzymatic antioxidants include AsA, GSH, Pro, carotenoids, flavonoids, and tocopherol (Yang and Guo, 2018b; Liu et al., 2022).

Superoxide dismutase is the most effective ROS scavenger and converts O_2_^⋅–^ to H_2_O_2_, which is further detoxified to H_2_O by APX, CAT, and GSH-Px (Yang and Guo, 2018a). Based on the difference of metal cofactors and subcellular distribution, SODs are mainly categorized as copper/zinc SODs (Cu/Zn-SODs), iron SODs (Fe-SODs), and manganese SOD (Mn-SODs). Fe-SODs are mainly distributed in the chloroplast and cytoplasm, Mn-SODs in mitochondria, and Cu/Zn-SODs in chloroplasts, cytosol and peroxisomes (Czarnocka and Karpiñski, 2018). There are at least nine *SOD* genes in tomato, including four *Cu/Zn-SODs*, three *Fe-SODs* and one *Mn-SOD*, and most of them are regulated by salt stress (Feng et al., 2016). CAT rapidly catalyzes H_2_O_2_, producing H_2_O and O_2_. The expression of tomato *CAT* gene is fine-tuned under salt stress (Hernández-Hernández et al., 2018). Recently tomato microRNA398b (miR398b) is found to regulate antioxidant system under salt conditions. The inhibition of *Cu/Zn-SOD 1* (*CSD1*) expression and SOD activity caused by miR398b promotes the accumulation of O_2_^⋅–^. Meanwhile, miR398b also decreases the activity of APX and CAT, and the contents of GSH as well, leading to H_2_O_2_ accumulation (He et al., 2021).

The AsA-GSH recycling pathway, also known as Asada-Halliwell pathway comprises of AsA, GSH, and four enzymes (including APX, MDHAR, DHAR, and GR) (Figure 2). AsA-GSH pathway is the heart of antioxidant defense, which mainly detoxify the H_2_O_2_ (Hasanuzzaman et al., 2019). The first step of this pathway is catalyzed by APX, which converts H_2_O_2_ to water and monodehydroascorbate radical (MDHA) using AsA as the electron donor. The combination of salinity (100 mM NaCl) and heat (42°C; 4 h/day) stress greatly enhanced the activity of APX to detoxify H_2_O_2_ and prevent oxidative damage in tomato plants (Sousa et al., 2022). MDHAR catalyzes the reduction of primary oxidation MDHA to AsA, thus maintaining the AsA pool. Overexpression of tomato chloroplast *MDHAR* elevates the AsA levels, sustains APX activity and accelerates the reductive detoxification of H_2_O_2_, therefore enhancing the tolerance to osmotic stress induced by salt and polyethylene glycol (PEG) (Li et al., 2012). The PA mediator transglutaminase (TGase) increases the PAs accumulation, that further promotes the activity of antioxidant enzymes (SOD, APX, MDHAR, and CAT), which reduces salt-induced oxidative damage in tomato (Zhong et al., 2020). The powerful antioxidant system of wild tomato mitigates salt-induced oxidative damage by the high expression of defense genes with the enhanced activities of antioxidant enzymes (Kashyap et al., 2020a). Through DHAR, GSH converts the oxidized form dehydroascorbate (DHA) to the reduced form AsA, yielding oxidized glutathione (GSSG). Finally, GSSG is reduced to GSH by GR using NADPH as the electron donor (Hao et al., 2021). Both GSH-Px and GST use GSH pool to detoxify H_2_O_2_ by catalyzing its conjugation with GSH. In tomato chloroplasts, exogenous GSH alleviates salt stress by improving the content of endogenous GSH, GSH/GSSH ratio and activities of H_2_O_2_-scavenging enzymes, but the detailed mechanism of GSH-induced salt stress amelioration and detoxification awaits further study (Zhou et al., 2018).

#### Non-enzymatic scavenging system

The already mentioned AsA and GSH, as well as Pro, carotenoids, flavonoids, and tocopherol, are non-enzymatic antioxidants that play a role in ROS detoxification and retrograde signaling (Czarnocka and Karpiñski, 2018). AsA provides electrons for various antioxidant defense reactions, and participates in redox signal transduction and enzymatic activity regulation. The AsA accumulation enhances oxidative stress tolerance in tomato (Li et al., 2019c), and the inhibition of AsA oxidase increases AsA content and salt tolerance in cherry tomato (Abdelgawad et al., 2019). Seed priming with AsA enhances salt tolerance by modulation of antioxidant mechanisms (Alves et al., 2021). GSH plays an important antioxidant role as the reductant of ROS and the substrate for some peroxidase. Exogenous GSH increases tomato resistance to salt-induced oxidative stress by maintaining the homeostasis of cellular redox, cellular ion, and PAs (Zhou et al., 2017, 2019). Tocopherol can effectively scavenge ROS and lipid free radicals, and is a protector of biological membranes. Tocopherol in chloroplasts of tomato functions as an antioxidant under the moderate stress (50 mM NaCl) similarly as in the early phase of severe stress (150 mM NaCl for 2 days). While in the late phase of severe stress (150 mM NaCl for 5 days), it may be involved in senescence signaling pathway (Skłodowska et al., 2009). Carotenoids are essential components of the photosynthetic antenna and reaction center complex, and exhibit antioxidant activity by protecting the photosynthetic machinery. The key carotenoid-related genes and carotenoid biosynthesis in mature-green fruits of tomato are enhanced by salinity (Leiva-Ampuero et al., 2020). Flavonoids are thought to be antioxidants in photoprotection. Chloroplast-located flavonoids scavenge ^1^O_2_ under stress and inhibit cellular lipid peroxidation (Czarnocka and Karpiñski, 2018). The total flavonoid content is positively correlated with the salt tolerance of tomato, denoting the underlying role of flavonoids for enhancing salt tolerance (Alam et al., 2021). The osmotic regulator Pro is also an antioxidant with the ability of scavenging free radicals and inhibiting lipid peroxidation. The foliar spray of low concentration of Pro significantly increases Pro and total soluble protein contents and the glutamine synthetase activity, and enhances the salinity tolerance of tomato under field conditions (Kahlaoui et al., 2018). The Pro and AsA pathways synergistically maintain cellular redox homeostasis in tomato plants under the combination of salinity and heat (Lopez-Delacalle et al., 2021).

Phytohormones play important roles in salt stress-induced ROS signaling and scavenging pathways. Exogenous ABA promotes the accumulation of Pro and soluble sugar, reduces the content of ROS, and improves the ability of the antioxidant enzyme system in tomato plants under saline-alkaline stress (Xu et al., 2022). SA decreases the tomato mitochondrial hexokinases (*HXKs*) transcription and activity, which contributes to mitochondrial ROS production (Poór et al., 2019). The ROS accumulation is directly controlled by ET signaling triggered by salt stress. However, ET cannot influence the ROS generated by SA in tomato cell suspension (Poór et al., 2013). Brassinolide (BR)-induced H_2_O_2_ generation can stimulate ET biosynthesis, and ET can also promote H_2_O_2_ generation. While ET signaling pathway participates in BR-induced salt tolerance in tomato by reducing oxidative damage and enhancing antioxidant enzyme capacity (Zhu et al., 2016). Defective jasmonic acid (JA) synthesis in tomato plants under salt stress is associated with the reduced activity of enzymatic and non-enzymatic antioxidants (Abouelsaad and Renault, 2018). The roles of cross-talk among phytohormones in salt stress-induced ROS signaling and scavenging pathways in tomato need to be further investigated.

Gamma-aminobutyric acid metabolic pathway, also known as GABA shunt, is probably involved in salt tolerance of tomato by modulating amino acid synthesis and ROS metabolism. Exogenous GABA has positive effects on alleviating salt stress, which is mainly due to induced osmotic regulation and antioxidant metabolism by the salt- and exogenous GABA-induced endogenous GABA (Wu et al., 2020; Figure 2). In GABA shunt, glutamate decarboxylase (GAD) catalyzes the decarboxylation of glutamate to GABA, GABA transaminase (GABA-T) converts GABA to succinic semialdehyde (SSA), and SSA dehydrogenase (SSADH) catalyzes the oxidation of SSA to succinate. The enhanced ROS accumulation and increased salt sensitivity in *GADs*- and *GABA-Ts*-silenced tomato plants are possibly attributed to the impaired function of mitochondrial electron transport chain caused by the decreased succinate. However, *SSADH*-silenced plants exhibited less sensitiveness to salt stress, that may possibly due to the similar levels of ROS between silenced and control plants under salt stress (Bao et al., 2015).

### Epigenetic regulation

Epigenetic modifications, also known as chromatin modifications, contain DNA methylation, RNA-directed DNA methylation (RdDM), and histone modifications (Chinnusamy and Zhu, 2009; Deinlein et al., 2014). Epigenetic modifications regulate stress-responsive gene expression and plant development, conferring stress adaptation (Chinnusamy and Zhu, 2009; Figure 2).

DNA methylation is primarily catalyzed by the DNA methyltransferases (MTases) family. A total of 9 tomato MTases genes are identified, including 1 methyltransferase (MET) member (*MET1*), 3 chromomethylase (CMT) members (*CMT2*, *CMT3*, and *CMT4*), 4 domains rearranged methyltransferase (DRM) members (*DRM5*, *DRM6*, *DRM7*, and *DRM8*), and 1 DNA methyltransferase homolog 2 (DNMT2) member (*METL*). Except for *MET1* and *DRM8*, all other MTase genes are significantly regulated by salt stress, suggesting that tomato MTase genes may be involved in salt stress response (Guo et al., 2020). *PKE1* is a Pro-, lysine-, and glutamic-rich protein gene, and the lower expression level in tomato leaves is consistent with the hypermethylation of its coding sequence. Tomato PKE1 confers the salt tolerance involved in post-transcriptional regulation through binding to F-box proteins (Li et al., 2019a). The functions of heavy methylation in the promoter of *PKE1* in tomato fruit and leaf salt responses remain unclear (Li et al., 2019a; Liu and He, 2020).

Small RNAs play the pivotal role in environmental stress responses of crop plants by regulating gene expression, and their generation mainly depends on proteins encoded by respective Dicer-like (DCL), Argonaute (AGO), and RNA-dependent RNA polymerases (RDR) gene families (Huang et al., 2016). *Arabidopsis* AtAGO4 is required for RdDM of the *SUPERMAN* (*SUP*) locus (Zilberman et al., 2003). In tomato, a total of 15 AGO proteins are identified, and 4 members are orthologs of AtAGO4, namely AGO4A-AGO4D (Xian et al., 2013). Tomato *AGO4A* is significantly induced by salt and drought stress (Bai et al., 2012). Down-regulation of *AGO4A* conferred enhanced salt and drought tolerance in transgenic tomato by reducing the transcript levels of DNA MTase genes (*DRMs*) and RNA silencing pathway genes, suggesting that tomato *AGO4A* as a core factor of RdDM pathway plays a negative role under salt and drought stress probably through regulating methylation process-associated genes (Huang et al., 2016).

In general, histone modifications are associated with changes in stress-induced gene regulation (Chinnusamy and Zhu, 2009). Histone post-translational modifications (HPTMs) include acetylation and methylation, acetylation is dynamically regulated by histone acetylases (HATs) and histone deacetylases (HDACs), and methylation is balanced by the activities of histone methylases (HMTs) and histone demethylases (HDMs). Plant HDACs are divided into three subfamilies: RDP3/HDA1 (Reduced Potassium Dependence 3/Histone Deacetylase 1, hereinafter named HDAs), plant-specific HD2s (Histone Deacetylase 2), and SIR2 (Silent Information Regulator 2) (Guo et al., 2017), and the members of HDA subfamily share sequence homology in the HDAC domain and require the Zn^2+^ cofactor for deacetylase activity (Yang and Seto, 2007; Zhao et al., 2015). There are 124 histone modifiers (HMs) in tomato, including 32 HATs, 14 HDACs (9 HDAs, 3 HD2s, and 2 SIR2s), 52 HMTs, and 26 HDMs (Aiese Cigliano et al., 2013). Among nine tomato *HDA* genes (named as *HDA1*-*HDA9*), the expression levels of *HDA1*, *HDA4*, and *HDA9* in root and that of *HDA3* in leaf were significantly stimulated by slat treatment, and the levels of *HDA2*, *HDA5*, and *HDA6* were induced both in roots and leaves (Guo et al., 2017). The *HDA5-*silenced tomato plants exhibited reduced tolerance to salt stress (Yu et al., 2018), while the stress-related genes modified by HDA5 through histone deacetylation under salt conditions remains to be identified.

Not only HDACs but also HATs are implicated in regulation of salt tolerance. In *Arabidopsis*, HAT general control non-repressed protein 5 (GCN5)-mediated acetylation of lysine 9 (H3K9) and lysine 14 of histone H3 (H3K14) is associated with activation of cellulose synthesis genes *CTL1* (chitinase-like gene), *PGX3* (*polygalacturonase involved in expansion-3*) and *MYB54* (*MYB domain protein-54*) under salinity. The severe growth inhibition and defects in cell wall integrity phenotypes of *Arabidopsis gcn5* mutant under salt stress can be partially rescued by overexpression of chitinase-like protein *CTL1* and can be almost fully recaptured by constitutive wheat *TaGCN5* expression. GCN5 enhances salt tolerance through activating cellulose synthesis genes and GCN5-mediated salt tolerance may be conserved between monocot and dicot plants (Zheng et al., 2019). Tomato GCN5 can catalyze acetylation on histone H3 at H3K9 and H3K14 residues and constitutive *GCN5* expression almost fully rescues growth defect phenotype of *Arabidopsis* null-mutant *gcn5-7*, indicating that tomato GCN5 functions similarly as AtGCN5 in developmental processes (Hawar et al., 2022). Nonetheless, the function of *GCN5* in tomato salt tolerance requires further elucidation.

### Gene expression changes

Plants generate salt tolerance mechanisms to minimize the adverse effects of salt stress by regulating the expression of salt response genes. Comprehensive analysis of gene expression profiles under salt stress by transcriptome technology can provide key clues for elucidating the molecular mechanism of salt stress in tomato. However, genes involved in salt tolerance may occur in wild tomato species but not in cultivated species (Foolad, 2007).

The wild tomato genotype *S. pimpinellifolium* ‘PI365967’ is more salt tolerant than the cultivar *S. lycopersicum* ‘Moneymaker’ (Sun et al., 2010). After treatment with 200 mM NaCl for 5 h, the number of differentially expressed genes (DEGs) in Moneymaker (1386) was higher than that in PI365967 (948). Eighty-six genes were specifically up-regulated in PI365966, including the genes encoding salicylic acid-binding protein 2 (SABP2), CIPKs, plasma membrane ATPase 1, peroxidase, lactoylglutathione lyase/glyoxalase I, and TINY-like protein and AP2/ERF domain protein (both belonging to DREB TF superfamily). Additionally, several genes encoding glutathione S-transferase showed significantly higher basal expression in PI365967 than in Moneymaker. This suggests that multiple strategies, such as SA signaling, SOS pathway, transcriptional regulation, ROS scavenging and detoxification synergistically confer salt tolerance in wild tomato. Among the 82 genes specifically down-regulated in PI365967, the gene encoding putative high-affinity nitrate transporter (a repressor of lateral root initiation) was most down-regulated (8-fold), which may promote root growth in PI365967 under salt stress conditions (Sun et al., 2010).

Similarly, another report using transcriptome analysis to reveal salt tolerance mechanisms found that most of the upregulated DEGs were involved in catalysis, transcriptional regulation and molecular transduction in wild tomato *S. chilense* under 500 mM NaCl conditions. However, the down-regulated DEGs were mainly involved in binding, molecular function regulator and catalysis (Kashyap et al., 2020b). Specifically, genes actively involved in Pro and arginine metabolism, oxidoreductase activity, hormone metabolism, ROS scavenging systems, signaling regulation, transporters, osmotic regulation, defense and stress responses, homeostasis and TFs are significantly up-regulated, and play an important role in salt tolerance of *S. chilense*. Genes encoding pentatricopeptide repeat-containing protein in *S. chilense* are significantly down-regulated, possibly defending against salt stress. Interestingly, although the existence of *Wnt* signaling (Wingless-related integration site) in plant systems is unknown, it plays a crucial role in conferring salt tolerance in *S. chilense* (Kashyap et al., 2020b). Transcriptome analysis of *S. lycopersicum* cv. MicroTom under salt (150 mM NaCl for 6 h) and oxidative stress (20 mM H_2_O_2_ for 6 h) revealed 6,643 significantly DEGs, including 3,950 DEGs identified under oxidative stress and 4,617 DEGs found under salt stress (Keshishian et al., 2018). Whereas up to 67.6% (2557) of induced and 75.6% (2162) of repressed DEGs show unique stress (salt or oxidative stress) regulation. Of the 33 cytokinin-related DEGs that were significantly regulated by stress, only 10 were regulated by both oxidative and salt stress, suggesting that although there is crosstalk between salt and oxidative stress, the transcriptional patterns of their gene regulation are not identical (Keshishian et al., 2018). Overall, the comparative transcriptomic analysis of tomato cultivars with different salt tolerance can help to improve our understanding on possible molecular mechanisms underlying salt tolerance in tomato.

## Approaches for improving salt tolerance of tomato

### Genetic engineering

Salt-tolerant transgenic lines have been developed for different metabolic properties, such as ion transport, osmoregulation, antioxidants, stress proteins, and universal stress proteins (TFs and signal transduction) (Ashraf and Munns, 2022). A series of tomato TFs are used to regulate salt tolerance via genetic engineering (Table 1). Overexpression of C2H2 zinc-finger protein *ZFs* improves salt tolerance by maintaining photosynthesis, increasing PA biosynthesis, and improving the ability of antioxidant AsA-mediated removal of ROS (Hichri et al., 2014; Li Y. et al., 2018). *bZIP1*, a member of the basic region/leucine zipper (bZIP) family, positively regulates salt tolerance by modulating ABA-mediated signaling pathways (Zhu et al., 2018). Overexpression of basic helix-loop-helix (bHLH) transcription factor *bHLH22* enhances the resistance to salinity and drought by increasing osmotic potential, augmenting the accumulation of flavonoids and ABA, and improving the active oxygen scavenging system (Waseem et al., 2019). Tomato plants overexpressing *WRKY8* improves salinity tolerance through enhancing the transcriptional levels of stress-responsive genes, Pro accumulating and activities of ROS-scavenging enzymes (Gao et al., 2020). Overexpressing *MYB102* confers salt tolerance by regulating Na^+^/K^+^ homeostasis, ROS scavenging ability, and expression of salt stress-related genes (Zhang et al., 2020b). NAC transcription factor *TAF1* increases the accumulation of Pro and Na^+^ ions in shoots, upregulates salt stress-responsive and ABA biosynthesis genes, and reduces stomatal conductance and stomatal pore area, thereby conferring salt tolerance (Devkar et al., 2020).

**TABLE 1:** Summary of genes involved in salt stress in tomato.

Gene name	Description	Function	References
*Salt Overly Sensitive 1 (SOS1)*	PM Na^+^/H^+^ antiporter	Maintained ion homeostasis, prevented Na^+^ from reaching photosynthetic tissues; natural variations in cultivated tomato increased salt sensitivity	Olías et al., 2009a; Wang Z. et al., 2021
*Salt Overly Sensitive 2 (SOS2)*	Calcineurin-interacting protein kinase	Increased salinity tolerance via regulating Na^+^/H^+^ and (Na^+^, K^+^)/H^+^ transporters responsible for cell ion homeostasis	Huertas et al., 2012
*Na^+^/H^+^ antiporter 2 (NHX2)*	K^+^/H^+^ antiporter	Conferred salt tolerance by improving K^+^ homeostasis and compartmentalization, and through joint overexpressing with *SOS2*	Rodríguez-Rosales et al., 2008; Huertas et al., 2013; Baghour et al., 2019
*DNA-binding with one finger 22 (Dof22)*	TF with C2-C2 zinc finger	Suppressing *Dof22* increased the levels of AsA to 1.33- and 1.64-fold in leaves and ripe fruits, respectively; decreased chlorophyll content by 77∼80% under salt stress; significantly reduced the fresh weight after salt treatment; slightly induced the expression levels of antioxidant related genes (from 1.5- to 2-fold); significantly down-regulated *SOS1*, obviously induced *NHX1* and *NHX2*; Dof22 could bind to the promoter of *SOS1* in yeast	Cai et al., 2016
*Basic region/leucine zipper 1 (bZIP1)*	TF with basic leucine zipper	*bZIP1*-RNAi transgenic plants exhibited reduced salt tolerance, decreased ABA and chlorophyll content and CAT activity, increased MDA content, and downregulated transcription levels of multiple genes encoding defense proteins related to abiotic stress and biotic stress	Zhu et al., 2018
*Basic region/leucine zipper 38 (bZIP38)*	Basic leucine zipper TF	Overexpression of *bZIP38* significantly decreased salt tolerance in tomato, reduced the chlorophyll by 50% and free Pro content by 25% in leaves, but increased the MDA content (from 1.5- to 2-fold); bZIP38 is a negative regulator of salt resistance that acts by modulating ABA signaling	Pan et al., 2017
*S-adenosylmethionine synthetase 1 (SAMS1)*	S-adenosylmethionine synthetase	Overexpression of *SAMS1* improved salt tolerance, significantly enhanced water-retention capacity and photosynthetic capacity, reduced the accumulation of superoxide, H_2_O_2_ and MDA, and enhanced ABA content and ROS scavenging enzymes activities; modulated the generation of PAs and H_2_O_2_ to maintain a better water homeostasis; reduced water loss under ABA treatment	Zhang et al., 2020a
*microRNA398b (miR398b)*	Conserved miRNA regulated CSD transcription	Overexpression of *miR398b* increased the sensitivity to salinity, enhanced the oxidative stress via the accumulation of O_2_^⋅–^, induced photoinhibition and inhibited the photosynthesis under salinity; miR398b regulated the expressions of antioxidant genes, activity of antioxidant enzymes and contents of antioxidants	He et al., 2021
*High-affinity K^+^ 20 (HAK20)*	Na^+^/K^+^ transporter	HAK20 transported Na^+^ and K^+^ and regulated Na^+^ and K^+^ homeostasis under salt conditions; a variation in the coding sequence of *HAK20* was associated with Na^+^/K^+^ ratio and conferred salt tolerance in tomato; knockout mutations in *HAK20* resulted in hypersensitivity to salt stress	Wang et al., 2020
*Basic helix-loop-helix 22 (bHLH22)*	TF with a basic helix-loop-helix domain	Plants overexpressing *bHLH22* showed short height with small leaves and enhanced flavonoid accumulation; overexpressing *bHLH22* displayed an enhanced tolerant to salinity, significantly peaked the activities of CAT, SOD, and POD to minimize the impacts of ROS such as H_2_O_2_	Waseem et al., 2019
*SALT TOLERANCE ENHANCER1 (STE1)*	Protein without any known conserved domains	Overexpression of *STE1* enhanced the tolerance to multiple chloride salts and oxidative stress, along with elevated antioxidant enzyme activities, increased ABA and chlorophyll contents, reduced MDA and ROS accumulations, decreased K^+^ efflux and increased H^+^ efflux; *STE1*-RNAi plants displayed the decreased salt tolerance; *STE1*-overexpression plants showed the increased sensitivity to ABA; STE1 promoted ABA-dependent salt stress-responsive pathways by interacting with PYLs and SnRK2s	Meng et al., 2020
*WRKY 3 (WRKY3)*	TF with conserved WRKYGQK domain	Overexpression of *WRKY3* reduced oxidative stress and Pro content under salt conditions, decreased Na^+^ content in leaves, induced accumulation of K^+^ and Ca^2+^, and up-regulated genes coding for antioxidant enzymes, ion and water transporters, or plant defense proteins	Hichri et al., 2017
*WRKY 8 (WRKY8)*	TF with conserved WRKYGQK domain	Overexpression of *WRKY8* in tomato displayed the alleviated wilting or chlorosis phenotype under salt stresses, with higher levels of stress-induced osmotic substances like Pro and higher transcript levels of the stress-responsive genes *AREB*, *DREB2A* and *RD29*	Gao et al., 2020
*WRKY 39 (WRKY39)*	TF with conserved WRKYGQK domain	Enhanced salt tolerance in tomato via accumulating Pro, reducing MDA, and up-regulating the expression of *RD22* and *DREB2A*	Sun et al., 2015
*HD-Zip homeobox 2 (HB2)*	TF with conserved HD and leucine zipper domains	*HB2*-RNAi transgenic plants increased the levels of chlorophyll and water content, reduced water loss rate and MDA content in the leaves, enhanced tolerance to salt stress; HB2 acted as a negative regulator in the high-salinity stress signaling pathways	Hu et al., 2017
*Caffeic Acid O-Methyltransferase 1 (COMT1)*	Critical enzyme for melatonin synthesis	Increased melatonin level and salt tolerance, maintained balance of Na^+^/K^+^, decreased ion damage, enhanced antioxidant capability, and up-regulated stress-related genes	Liu et al., 2019; Sun et al., 2020
*DEFENSELESS1 (DEF1)*	Protein involved in JA synthesis	Mutation of *DEF1* decreased nitrogen content in both leaves and roots, repressed the activity of both enzymatic antioxidants and non-enzymatic antioxidants; *def-1* plants exhibited oxidative stress symptoms and ionic imbalance	Abouelsaad and Renault, 2018
*Glyoxalase I (GlyI) and Glyoxalase II (GlyII)*	Two enzymes catalyzed conversion of methylglyoxal to D-lactic acid	The transgenic lines overexpressing *GlyI* and *GlyII* under a high NaCl concentration (800 mM) showed reduced lipid peroxidation and the production of H_2_O_2_ in leaves, and a lower decrease in the content of chlorophyll a + b	Viveros et al., 2013
*MADS-box protein 8 (MBP8)*	TF with typical MADS domain region in N-terminus	The *MBP8*-RNAi transgenic plants were less inhibited by salt at post-germination stage, improved tolerance to stress, displayed the higher levels of chlorophyll and water content, lower water loss rate and MDA content, and significantly up-regulated the expression of multiple stresses related genes; MBP8 functioned as a negative stress-responsive TF in the high salinity stress signaling pathways	Yin et al., 2017
*MADS-box protein 11 (MBP11)*	TF with typical MADS domain region in N-terminus	*MBP11-*RNAi plants were less tolerance to salt stress, decreased relative water and chlorophyll content, and increased relative electrolyte leakage and MDA content; overexpression of *MBP11* enhanced salt tolerance; MBP11 acted as a stress-responsive TF in the positive modulation of salt tolerance	Guo et al., 2016
*Argonaute 4A (AGO4A)*	Core factor of RdDM pathway	*AGO4A*-down-regulating transgenic plants showed enhanced tolerance to salt and drought stress; the expression levels of some DNA methyltransferase genes and RNAi pathway genes were significantly lower in *AGO4A*-down-regulating plants than in WT plants; *AGO4A* plays a negative role under salt stress probably through the modulation of DNA methylation as well as the classical RNAi pathway	Huang et al., 2016
*Transcription Activation Factor 1 (TAF1)*	TF with highly conserved NAM domain	Overexpression of *TAF1* improved salinity tolerance, lowering *TAF1* expression caused stronger salinity-induced damage; shoots of *TAF1* knockdown plants accumulated more toxic Na^+^ ions; in *TAF1* knockdown plants during salinity stress, stomatal conductance and pore area were increased, salinity-induced changes in tricarboxylic acid cycle intermediates and amino acids are more pronounced, and Pro accumulation was decreased; TAF1 controls the tomato’s response to salinity stress by combating both osmotic stress and ion toxicity	Devkar et al., 2020
*Zinc Finger 2 (ZF2)*	C2H2 zinc finger TF with ERF-associated amphiphilic repression domain	Tomato ZF2 enhanced salt sensitivity in *Arabidopsis*, whereas delayed senescence and improved salt tolerance in tomato, particularly by maintaining photosynthesis and increasing PA biosynthesis; *ZF2* is rapidly induced by ABA treatment, and tomato overexpressing *ZF2* accumulated more ABA than WT plants	Hichri et al., 2014
*Zinc-finger protein 3 (ZF3)*	TF with EAR motif	Overexpression of *ZF3* significantly increased the levels of AsA in tomato and *Arabidopsis* and the AsA-mediated ROS-scavenging capacity, enhanced the salt tolerance; ZF3 directly bound CSN5B and this interaction inhibited the binding of CSN5B to VTC1, a GDP-mannose pyrophosphorylase; the EAR domain promoted the stability of ZF3; ZF3 simultaneously promoted the accumulation of AsA and enhanced salt tolerance	Li Y. et al., 2018
*RING (Ring)*	E3 ubiquitin ligase with RING finger conserved region	Ring localized at the endoplasmic reticulum; Silencing of *Ring* increased the sensitivity to salt stress in wild tomato; Overexpression of *Ring* in *Arabidopsis* resulted in enhanced salt tolerance during seed germination and early seedling development; Ring functioned as a positive regulator of salt tolerance	Qi et al., 2016
*NAM, ATAF and CUC transcription factor 35 (NAC35)*	TF with conserved NAC domain and diversified C-terminal region	Overexpression of *NAC35* promoted root growth and development under salt stress, induced higher expressions of *ARF1*, *ARF2*, and *ARF8* in transgenic lines	Wang et al., 2016
*Hybrid proline-rich protein 1 (HyPRP1)*	Putative plant cell wall glycoprotein with repetitive Pro-rich N-terminal domain and conserved eight-cysteine motif C-terminal domain	*HyPRP1*-RNAi transgenic plants enhanced tolerance to various abiotic stresses (oxidative stress, dehydration, and salinity); SO_2_ detoxification-related enzymes, including sulfite oxidase, Fds, and Msr A, interacted with HyPRP1; more sulfates and transcripts of *Msr A* and *Fds* were accumulated in *HyPRP1* knockdown lines exposed to SO_2_ gas; HyPRP1 was a negative regulator of salt and oxidative stresses and was probably involved in sulfite metabolism	Li et al., 2016
*Altered response to salt stress 1 (ARS1)*	R1-type MYB TF with conserved MYB-like and adjacent P-rich domains	The *ars1* mutant reduced fruit yield under salt acclimation; the stomatal behavior of *ars1* mutant leaves induced higher Na^+^ accumulation via the transpiration stream; the mutation affected stomatal closure in a response mediated by ABA; *ARS1* contributed to reduce transpirational water loss under salt stress	Campos et al., 2016
*v-myb avian myeloblastosis viral oncogene homolog 49 (MYB49)*	TF with two highly conserved HTH DNA-binding domains	Overexpression of *MYB49* decreased the accumulation of ROS, MDA content, and relative electrolyte leakage, and increased POD activity, SOD activity, chlorophyll content, and photosynthetic rate under salt stress, improving salt tolerance	Cui et al., 2018
*v-myb avian myeloblastosis viral oncogene homolog 102 (MYB102)*	TF with two highly conserved HTH DNA-binding domains	Overexpression of *MYB102* maintained a better K^+^/Na^+^ ratio, lower ROS and lower electrolytic leakage rates, increased accumulation of antioxidants and Pro, and upregulated transcripts of salt stress-related genes	Zhang et al., 2020b
*BRASSINAZOLE RESISTANT 1 (BZR1)*	TF with nuclear localization sequence, PEST domain, and BIN2 phosphorylation domain	Overexpression of *BZR1D* enhanced the BR response and improved salt tolerance in *Arabidopsis*; *BZR1D*-overexpressing tomato lines showed a short plant height, smaller and curly leaves, and delayed flowering; BZR1D positively regulated salt tolerance in tomato and upregulated the expression of multiple stress-related genes	Jia et al., 2021
*Histone deacetylase A5 (HDA5)*	Histone deacetylase protein with deacetylase catalytic domain	Seedlings growth of *HDA5*-RNAi plants were more inhibited on the medium containing salt compared with WT; under salt stress, chlorophyll in mature leaves degraded earlier in transgenic leaves, and transgenic plants displayed wilting earlier and more severe than WT; silencing of *HDA5* resulted in decreasing tolerance to salt	Yu et al., 2018
*Heat stress transcription factor 3 (HsfA3)*	TF with highly conserved DBD, OD, and putative NLS	HsfA3 played a negative role in controlling seed germination under salt stress; in the presence of 120 mM NaCl, half of the WT seeds were successfully germinated on the second day after stratification, whereas the germination percentages of *HsfA3*-overexpressing seeds were less than 10%	Li Z. et al., 2013
*CALCINEURIN B-LIKE PROTEIN 10 (CBL10)*	EF (helix-loop-helix structural motif)-hand Ca^2+^ protein sensor	Lack function of CBL10 leaded to the severe damage in the shoot apex and reproductive organs under salinity conditions; CBL10 mediated salt tolerance by regulating Na^+^ and Ca^2+^ fluxes in the vacuole, cooperating with the vacuolar cation channel TPC1 and the two vacuolar H^+^-pumps, AVP1 and VHA-A1, which in turn were potential targets of CBL10	Egea et al., 2018
*Tomato 14-3-3 Protein 7 (TFT7)*	Phosphoserine-binding protein with conserved target binding domain	Transgenic plants overexpressing *TFT7* improved germination rate, dry mass, total chlorophyll concentration and root length under salt stress; the degree of H_2_O_2_ and MDA accumulation were inhibited in transgenic plants; TFT7 upregulated the activity of APX that played the indispensable role in salt-induced oxidative stress	Xu and Shi, 2007

ABA, abscisic acid; APX, ascorbate peroxidase; AsA, ascorbic acid; BR, brassinolide; CAT, catalase; Fds, ferredoxins; MDA, malondialdehyde; Msr A, methionine sulfoxide reductase A; PAs, polyamines; PM, plasma membrane; POD, peroxidase; Pro, proline; RNAi, RNA interference; ROS, reactive oxygen species; SnRK2s, sucrose non-fermenting-1-related protein kinase 2s; SOD, superoxide dismutase; TF, transcription factor; TPC1, TWO-PORE CHANNEL 1; WT, wild type.

By genetic transformation, other genes involved in tomato oxidative stress and ion homeostasis have been shown to improve salt tolerance. RING finger E3 ligase *Ring* functions as a positive regulator of salt stress signaling through regulating the ion homeostasis of Na^+^ and K^+^, levels of H_2_O_2_ and lipid peroxidation, and expression of stress-related genes (Qi et al., 2016). Overexpression of the melatonin synthesis-related gene *Caffeic Acid O-Methyltransferase 1* (*COMT1*) increases salt tolerance via maintaining the balance of Na^+^/K^+^, decreasing ion damage by activating SOS pathway, enhancing the antioxidant capability, and upregulating stress-related genes (Liu et al., 2019; Sun et al., 2020). Small SALT TOLERANCE ENHANCER1 (STE1) protein promotes ABA-dependent salt stress-responsive pathways by interacting with the ABA receptor PYLs and the positive ABA signaling regulator SnRK2s and by improving Na^+^ and K^+^ homeostasis and ROS scavenging (Meng et al., 2020).

Heterologous overexpression of salt stress-related genes can also improve salt tolerance in tomato. For example, overexpressing yeast *trehalose-6-phosphate synthase* (*TPS1*) in tomato increases salt tolerance, which is partly due to the promotion of trehalose biosynthesis (Cortina and Culiáñez-Macià, 2005), and the enhanced salt tolerance in tomato overexpressing *Arthrobacter globiformis* choline oxidase *codA* is partly attributed to the accumulating GB (Wei et al., 2017).

Although transgenic technology can improve the salt tolerance of plants through a single strategy, the degree of improvement is limited due to the polygenic trait of salt tolerance (Hao et al., 2021). Promising achievements in improving salt tolerance may be achieved by stacking/aggregating multiple genes (Ashraf and Munns, 2022). Precise editing of multi-target genes using clustered regularly interspaced short palindromic repeats (CRISPR)/CRISPR-associated (Cas) technology has emerged as an alternative to traditional plant breeding and transgenic methods (Hanin et al., 2016). As an ideal candidate for CRISPR/Cas9 based gene modulations, tomato has already achieved salt tolerance regulation through genome editing (Chandrasekaran et al., 2021). For instance, both mutant alleles *slsos1-1* and *slsos1-2* generated by the CRISPR/Cas9 system increase the Na^+^/K^+^ ratio in tomato roots and the sensitivity to salt stress (Wang Z. et al., 2021). As a negative regulator of ABA, *hybrid proline-rich protein 1* (*HyPRP1*) plays a negative role in salt tolerance of tomato (Li et al., 2016). The precise elimination of HyPRP1 negative-response domain(s) by CRISPR/Cas protein-based targeted mutagenesis improves salinity tolerance in both germination and vegetative phases (Tran et al., 2021). The application of CRISPR/Cas in tomato also includes enhancing cultivation, promoting growth, alleviating biotic stress, and improving tolerance to abiotic stresses such as drought and temperature. This technology has a good prospect in the breeding and genetic research of tomato (Chandrasekaran et al., 2021). The in-depth understanding of the molecular mechanism of salt stress and the advancement of efficient and precise CRISPR/Cas technology are expected to accelerate the breeding of salt-tolerant tomato varieties with high yields.

### Marker-assisted selection

Since salt tolerance is a polygenic trait, traditional breeding methods to improve salt tolerance of crops are time-consuming and labor-intensive, and may introduce undesirable traits while selecting traits. The combination of QTL analysis and marker-assisted selection (MAS) is predicted to be an effective method for simplifying this process (Hanin et al., 2016). MAS can be used in the selection of lines following a crossing program and without the need to evaluate performance of plants under stress (Munns et al., 2002; Flowers and Flowers, 2005). Through association analysis of molecular markers and salt stress phenotypes can not only assess the molecular basis of tomato salt tolerance, but it also provides guidance for the introgression of salt tolerance traits in the target varieties (Gharsallah et al., 2016a; Ezin et al., 2018).

Many QTLs associated with tomato salt response (including plant height, stem diameter, leaf number, leaf and root fresh and dry mass, ion concentration, antioxidant response, and survival rate) have been identified (Foolad, 2007; Villalta et al., 2008; Frary et al., 2010, 2011; Asins et al., 2013). These QTLs may be useful in breeding of the salt tolerant cultivars. Nevertheless, the accuracy and precision of QTL identification, the complexity of genetic and environmental interactions and the lack of evaluation reports under field conditions greatly limit progress in marker-assisted breeding of salt tolerance (Ashraf and Foolad, 2013). Molecular linkage map and identification of salt tolerance related QTLs are the primary requisite for improving the salt tolerance in tomato through MAS and pyramiding, while the next generation sequencing (NGS) helps to obtain high density genetic maps, which promotes MAS to play a substantial role in the molecular breeding of salt-tolerant tomato varieties (Hanin et al., 2016; Kashyap et al., 2021).

Genome-wide association studies can overcome the limitations of traditional QTL mapping, provide higher mapping resolution, and detect multiple alleles at the same locus (Ashraf and Munns, 2022). GWAS has become a more efficient technique for studying genetics underlying trait variation (Morton et al., 2019). Valid phenotypic data is a prerequisite for gene/QTL discovery, association mapping and GWAS (Qin et al., 2020). A GWAS of the root Na^+^/K^+^ ratio trait in a tomato population comprising materials from different genetic backgrounds reveals that *HAK20* gene is effectively involved in the transport and maintenance of Na^+^ and K^+^ homeostasis under salt stress, thereby imparting salt tolerance to tomatoes (Wang et al., 2020). The model of high-throughput phenotype-genotype interaction will greatly facilitate the genetic dissection of salt tolerance-related traits in tomato, paving the way for the development of salt-tolerant lines/genotypes.

### Grafting

Grafting is an economically justified and sustainable strategy to overcome saline stress, and offers an alternative to breeding of salt-tolerant tomato (Singh et al., 2020; Table 2). The effect of grafting depends on the characteristics of the scion and rootstock, their interaction and stress intensity (Giordano et al., 2021). Grafting salt-susceptible tomato cultivars onto salt-tolerant rootstock can effectively reduce the adverse effects of salt stress on growth, yield, and fruit quality. The root system of grafted plants is stronger and more efficient in uptake of water and nutrients. In addition, grafted tomato plants improve salt tolerance by reducing ionic stress, increasing the transfer of K^+^, Ca^2+^ and Mg^2+^ to shoots and leaves, and reducing ROS-induced oxidative damage (Koleška et al., 2018; Singh et al., 2020). The salt resistant tomato rootstock greatly reduces the yield loss of the sensitive genotypes and increases fruit quality under saline conditions. Salt-tolerant rootstock also controls the stomatal openness and closure of sensitive scions, improves the osmotic adjustment of leaves, and reduces the transport of accumulation of Na^+^ ions accumulation to young leaves (Coban et al., 2020).

**TABLE 2:** Summary of eco-sustainable approaches to improve salt tolerance in tomato.

Approach	Effect	References
**Grafting**		
Tomato grafted tomato	Grafting the sensitive genotype onto tolerant genotype reduced the yield loss from 44 to 3%, increased fruit size, total dry matter content, and vitamin C, while decreased pH under saline conditions; the tolerant rootstock controlled sensitive scions’ stomatal openness and closure; the tolerant genotype ameliorated leaf osmotic adjustment of the sensitive genotype in grafting under salt stress, and decreased the transport of Na^+^ ions to young leaves in the grafting combination	Coban et al., 2020
Tomato grafted eggplant	Grafting improved tomato plant performance under salt stress, and eggplant rootstock IC-111056 outperformed IC-354557; compared with non-grafted control at EC 6 and 9 dS m^–1^, the increase in the average fruit yield of grafted plants with rootstock IC-111056 was 24.41 and 55.84%, respectively, and that with IC-354557 was 20.25 and 49.08%, respectively; grafted plants maintained a superior water status under saline irrigation along with higher Pro and antioxidant enzyme activities; rootstocks regulated the partitioning of toxic saline ions in the scions by promoting higher Na^+^ accumulation in the old leaves and lower in the young leaves of grafted plants	Sanwal et al., 2022
Tomato grafted potato	The grafted plants balanced mineral partitioning across plant parts; grafted plants were superior in water productivity by 56.8 and 70.5% over the control plants under saline and non-saline water-irrigations, respectively; potato rootstock improved the tolerance of tomato scion to saline water irrigation through distinct changes in dry mass allocation, and the induction of mineral-compartmentalization processes	Parthasarathi et al., 2021
Tomato grafted wolfberry	Grafting onto wolfberry increased the SPAD in tomato leaves under salt stress, remained the light use ability of the leaf chlorophyll in saline soil; tomato grafted onto wolfberry had significantly increased fruit fiber and soluble sugar concentration and reduced vitamin C concentration; the growth and fruit yield of the tomato grafted on wolfberry were reduced, but the union was not sensitive to salt stress	Feng et al., 2019
**Pretreatments**		
Heat treatment	Increased the accumulation of GB and trehalose, maintained a higher K^+^ level, with a better performance of cell water status and photosynthesis	Rivero et al., 2014
Salinity acclimation	Improved fruit quality, reduced the concentration of Na^+^ in leaves, accumulated Pro, and activated antioxidant enzymes	Kamanga et al., 2020; Meza et al., 2020
Elevated CO_2_	Enhanced growth, stimulated photosynthesis, reduced ABA and ET precursor, improved the antioxidant capacity, ion homeostasis and PA metabolism	Brito et al., 2020; Zhang et al., 2020c
Low red to far-red light ratio (R:FR)	Low R:FR significantly alleviated the damage of tomato seedlings from salt stress; On day 4, 8, and 12 at low R:FR, the Fv/Fm of PSII were increased by 4.53, 3.89, and 16.49%, respectively; the Pn of leaves were increased by 16.21, 90.81, and 118.00%, respectively; low R:FR enhanced the integrity and stability of the chloroplast structure through maintaining the high activities of antioxidant enzymes, mitigated the degradation rate of photosynthetic pigments caused by ROS, and upregulated the transcripts of antioxidative enzyme related genes, and enhanced salinity tolerance from the regulation of photosynthesis and ROS scavenging systems	Wang Z. et al., 2021
Vanillic acid and quercetin	Reduced Na^+^ content, increased LRWC and Pro, and reduced H_2_O_2_ and MDA content, and LOX activity; increased glutathione *S*-transferase activity in salt-invaded seedlings; caused the reduction of toxic methylglyoxal accumulation through the enhancement of glyoxalase system; promoted plant growth and photosynthetic pigments synthesis under salt conditions	Parvin et al., 2019a,^2020^
Spd and EBL	Exogenous Spd applied as a pre-soaking treatment to seeds promoted PA synthesis under salinity-alkalinity stress, and enhanced the salinity-alkalinity tolerance of tomato; EBL inhibited Na^+^ upward transport in flowers and apiculus of salt-stressed tomato, induced an obvious increase of PAs in young leaves, increased fruit-PAs concentration in mid-anaphase, and promoted the (Spd + spermine)/putrescine ratio in premetaphase of fruit period, improving salt resistance	Hu et al., 2012; Zheng et al., 2016
GSH	Improved photosystem II efficiency, balanced uneven distribution of light energy, enhanced antioxidant defense system, regulated synthesis and metabolism of GSH and PA, alleviated ion imbalance and poisoning	Zhou et al., 2019
Omeprazole	Improved growth, protected photosynthetic system, increased quantum yield of PSII, ABA, and Ca^2+^, decreased auxins, cytokinin, Na^+^, and Cl^–^	Rouphael et al., 2018
Vermicompost leachate	Improved growth, reduced Na^+^, decreased ET synthesis, increased Pro and anthocyanin, increased jasmonate, modified cytokinin profile	Benazzouk et al., 2020
Melatonin	Improved photosynthetic activities, enhanced antioxidant system, Pro and carbohydrates metabolism, improved osmoregulation	Yin et al., 2019
GABA	Reducing Na^+^ flux from root to leaves, increased amino acid content and strengthened antioxidant metabolism	Wu et al., 2020
Combined MEF	MEF-treatment significantly enhanced Pro accumulation in plants grown under 120 mM and 150 mM NaCl conditions, significantly improved nitrogen, phosphorus, and K^+^ absorption in plants grown at 80 mM and 120 mM NaCl levels, and significantly decreased leaf lipid peroxidation through ROS oxidative stress with enhanced CAT and SOD activities; MEF triggered a significant decline in fatty acid content, enhanced K^+^ uptake and reduced Na^+^/K^+^ ratio in the leaves of treated plants	Mutale-joan et al., 2021
**PGPB**		
Endophytic *Pseudomonas* spp. strain OFT5	Plants inoculated with the OFT5 strain inhibited the reductions in total biomass caused by salt stress, reduced salt-induced ET production, and promoted shoot uptake of macronutrients and micronutrients, which might activate processes that alleviate the effects of salt	Win et al., 2018
*Pseudomonas* 16S	Plants inoculated with *Pseudomonas* 16S showed higher biomass than both uninoculated and *Enterobacter* 15S inoculated plants under saline conditions, *Pseudomonas* 16S was efficient in alleviating the saline stress; *Pseudomonas* 16S induced an increase in the content of ROS-scavenging and antioxidant compounds in addition to the facilitation of Fe acquisition	Zuluaga et al., 2021
Endophytic halotolerant *Bacillus velezensis* FMH2	FMH2 treatment promoted plant growth in presence of salt stress, decreased endogenous Na^+^ accumulation and increased K^+^ and Ca^2+^ uptake; FMH2-treatment improved chlorophyll contents, membrane integrity and phenol peroxidase concentrations, and reduced MDA and H_2_O_2_ levels under saline conditions	Masmoudi et al., 2021
*Azotobacter chroococcum* 76A	The *A. chroococcum* 76A strain enhanced salinity tolerance in tomato; stress priming in plants inoculated with *A. chroococcum* 76A increased the expression of key stress-related genes; the application of optimal nutritional levels appeared to be inhibitory to the growth promoting and stress protective effects of *A. chroococcum* 76A	Van Oosten et al., 2018
*Pseudomonas oryzihabitans* AXSa06	Inoculations with *Pseudomonas oryzihabitans* AXSa06 repressed stress-inducing signals through a dampened ET and ABA metabolism and a reduced activation of downstream TFs when stress was applied; inoculations with AXSa06 alleviated the negative impact of salinity on photosynthetic machinery and carbon assimilation, through a more active ruBisCO and NR, involving an efficient mechanism of Na^+^ detoxification	Mellidou et al., 2021
**AMF**		
A mixture of *Glomus geosporum* and *Glomus intraradices*	Colonization of tomato roots with AMF significantly enhanced the reducing effect of salt stress on the transcription levels of tonoplast and PM aquaporin genes, and resulted in a dramatic increase in the mRNAs of three aquaporin genes in leaves under salt stress; AMF controlled the expression of aquaporins and thus might regulate water flow in tomato under salt stress	Ouziad et al., 2006
*Glomus mosseae*	AMF mitigated the adverse effects of salt stress, including reductions in root colonization, growth, leaf area, chlorophyll content, fruit fresh weight, and fruit yield; AM plants promoted P and K accumulation and reduced Na concentration; AMF colonization enhanced the activities of SOD, CAT, POD and APX in leaves, and reduced oxidative damage	Latef and Chaoxing, 2011
**Nanoparticles**		
Cu-NPs	The content of Cu increased in tomato plants under salinity with the application of Cu-NPs, which increased the phenols (16%) in the leaves and the content of vitamin C (80%), GSH (81%), and phenols (7.8%) in the fruit compared with the control; the enzyme activities of PAL, APX, GPX, SOD, and CAT increased in leaves by 104, 140, 26, 8, and 93%, respectively; foliar spraying of Cu-NPs on tomato plants under salinity appeared to induce stress tolerance to salinity by stimulating the antioxidant mechanisms	Pérez-Labrada et al., 2019
Cu-NPs + Cs-PVA	The application of Cs-PVA + Cu-NPs increased the stem diameter of tomato plants cultivated under non-stressed conditions; Cs-PVA + Cu-NPs increased plant height and stem diameter under salt conditions and induced the expression of the *SOD* and *JA* genes; the application of Cs-PVA and the Cu-NPs activated the antioxidant defense mechanisms and were mediated by the octadecanoid pathway of the jasmonates	Hernández-Hernández et al., 2018
ZnO-NPs	Foliar spray of ZnO-NPs significantly increased SL and RL, biomass, leaf area, chlorophyll content and photosynthetic attributes; ZnO-NPs mitigated the impacts of salt stress on tomato growth, and enhanced protein content and antioxidative enzyme activity such as POX, SOD and CAT; ZnO-NPs played an important role in the alleviation of salt toxicity in tomato plants	Faizan et al., 2021
Si-NPs + grafting	Foliar application of Si-NPs combined with grafting improved salt tolerance and reduced salt damage in tomato plants; plant growth, fruit yield, fruit quality, especially vitamin C content and TSS percentage, mineral content, and GA3, ABA, and Pro levels of grafted tomato combined with foliar application of Si-NPs were significantly higher than the self-grafted tomato under saline conditions	Sayed et al., 2022

AMF, arbuscular mycorrhizal fungi; Cs-PVA, chitosan-polyvinyl alcohol hydrogels; Cu-NPs, copper nanoparticles; DAS, days after sowing; EBL, 24-epibrassinolide; EC, electrical conductivity; ET, ethylene; Fv/Fm, maximum photochemical quantum yields; GA3, gibberellic acid; GABA, gamma-aminobutyric acid; JA, jasmonic acid; LOX, lipoxygenase; LRWC, leaf relative water content; MEF, microalgae-cyanobacteria extract formulations; NR, nitrate reductase; PAL, phenylalanine ammonia lyase; PGPB, plant growth promoting bacteria; Pn, net photosynthetic rates; POX, peroxidase; PSII, photosystem II; RL, root length; Si-NPs, silicon nanoparticles; SL, shoot length; SPAD, leaf chlorophyll index; Spd, spermidine; TSS, total soluble solids; ZnO-NPs, zinc oxide nanoparticles. The remaining abbreviations mentioned in this table exist in Table 1.

Intergeneric grafting is a promising approach for enhancing the salinity tolerance of tomato. Grafting on salt tolerant eggplant (*Solanum melongena*) rootstock increases the average fruit yield and improves salt tolerance of tomato scion. Grafted plants maintain higher relative water content and antioxidant enzyme activities, and balance the salt damage through accumulating Pro. Meanwhile, the eggplant rootstock confers Na^+^ exclusion and K^+^ retention properties to the tomato scion (Sanwal et al., 2022). Grafting tomato on potato (*Solanum tuberosum*) rootstock increases water productivity by 56.8% under saline water-irrigations, significantly alters dry matter allocation, and induces mineral compartmentalization processes (Parthasarathi et al., 2021). Although grafting tomato on halophyte wolfberry (*Lycium chinense*) can enhance salt tolerance, it reduces the growth and fruit yield of the grafted plants due to the limitation of thinner woody stem (Feng et al., 2019). In conclusion, the selection of suitable rootstocks is a prerequisite for getting the maximum benefit from grafting under salt stress.

### Pretreatments

Pretreatments increase the ability of tomato plants to adapt to salinity (Table 2). Seed priming and drought pretreatment have been reviewed elsewhere and will be not covered in this review (Cuartero et al., 2006).

Heat induces salinity tolerance of tomato through improving Na^+^ and K^+^ homeostasis, improving water balance, reducing oxidative stress, and increasing efficient photosynthetic performance (Rivero et al., 2014). Heat stress-induced salt tolerance belongs to a phenomenon known as cross-tolerance in plants. Thus, salinity can have a beneficial regulatory role in enhancing tomato tolerance to other stresses, such as protecting it against excessive sulfur toxicity (Jiang et al., 2017), significantly reducing the infection of biotrophic fungus *Oidium neolycopersici* (Achuo et al., 2006), and synergistically increasing the effect of DL-β-aminobutyric acid (BABA) on triggering systemic resistance in tomato plants against *Pseudomonas syringae* pv. *tomato* infection (Baysal et al., 2007). Salinity acclimation reduces the negative effects of salt stress (Kamanga et al., 2020), and moderate salt stress improves tomato fruit quality without decreasing yield (Meza et al., 2020). Elevated CO_2_ confers tomato tolerance to progressively higher soil salinity and secondary soil salinization by improving antioxidant capacity, ion homeostasis, and PA metabolism, decreasing ABA and ET levels, and suppressing transpiration (Yi et al., 2015; Brito et al., 2020; Zhang et al., 2020c). Furthermore, low red light (R) to far-red light (FR) ratio (R:FR) enhances salinity tolerance in tomato by regulating photosynthesis and ROS scavenging systems (Wang Y. et al., 2021).

Exogenous vanillic acid and quercetin improve salt tolerance by enhancing the action of glyoxalase system (Parvin et al., 2019a,^2020^). Spermidine and 24-epibrassinolide (EBR) enhance tomato tolerance to salinity and alkalinity stress by regulating PA metabolism (Hu et al., 2012; Zheng et al., 2016). Application of exogenous GSH reduces the level of PAs and promotes the transformation of PAs between different morphologies in tomato seedlings under salinity conditions, contributing to the improvement of salt tolerance (Zhou et al., 2019). Application of omeprazole (benzimidazole proton pump inhibitor) increases nutrient uptake and allocation, enhances photosynthesis and plant performance, thus improving resource use efficiency and salinity tolerance in tomato (Rouphael et al., 2018). Vermicompost leachate reduces the impact of salinity on leaf senescence and enhances salinity tolerance, which is related to the decreased ET synthesis, increased anthocyanin contents, and increased Pro and jasmonate accumulation (Benazzouk et al., 2020). The combined microalgae-cyanobacteria extract formulations (MEF) stimulates tomato plant growth and salt tolerance response through the enhanced antioxidant enzyme activities and the improved root growth and nutrient uptake (Mutale-joan et al., 2021).

Pretreatments may be simpler and more economical than other strategies to improve salt tolerance in tomato. The adaptive mechanism of tomato to salt stress can provide a theoretical basis for pretreatments to improve salt tolerance. The integration of metabolomics and other omics approaches will provide comprehensive insight into the response of tomato to salt stress.

### Modulation of the rhizosphere

Interaction with beneficial soil microorganisms improves salt tolerance (Hanin et al., 2016). As a product of the co-evolution between plants and microorganisms, plant growth promoting bacteria (PGPB) represent a new biological pathway for sustainable agriculture to alleviate salt stress (Singh et al., 2018). Inoculating tomato seedlings with endophytic *Pseudomonas* spp. strain OFT5 decreases salt-induced ET levels, but promotes shoot uptake of the macronutrients and micronutrients, improving plant growth under moderate salt conditions (Win et al., 2018). Tomato plants inoculated with *Pseudomonas* 16S showed higher biomass accumulation than uninoculated plants. It is the result of the facilitation of Fe acquisition and an increase in the content of ROS-scavenging and antioxidant compounds (Zuluaga et al., 2021). Endophytic halotolerant *Bacillus velezensis* FMH2 alleviates salt stress on tomato plants by regulating ion accumulation (decreased endogenous Na^+^ accumulation, increased K^+^ and Ca^2+^ uptake) and enhancing antioxidant responses (Masmoudi et al., 2021). Root inoculation with *Azotobacter chroococcum* 76A not only enhances tomato adaptation to salt stress under low nitrogen conditions, but also promotes nutrient assimilation efficiency under moderate and severe salinity, showing its potential in improved nutrition and salt stress protection (Van Oosten et al., 2018). Comparative transcriptomics and metabolomics reveal that *Pseudomonas oryzihabitans* AXSa06 mediates salt tolerance in tomato by efficiently activating antioxidant metabolism, by dampening stress signals, by detoxifying Na^+^, as well as by effectively assimilating carbon and nitrogen (Mellidou et al., 2021).

Arbuscular mycorrhizal fungi (AMF) can alleviate salt stress by enhancing assimilation and uptake of key elements, activating antioxidant systems and photosynthesis, regulating key hormone accumulation, and activating nutrient transporters and enzymes (Giordano et al., 2021). For instance, AMF colonization reduces the expression of aquaporins genes in roots of salt-treated tomato plants, whereas significantly increases their transcript levels in leaves, and thereby presumably regulates water flow in tomato under salt stress (Ouziad et al., 2006). Moreover, AMF colonization increases P and K concentration, decreases Na content, enhances the activities of SOD, CAT, POD and APX, and increases tomato fruit yield by 33.3 and 106% at 50- and 100-mM salinity levels, respectively (Latef and Chaoxing, 2011; Table 2).

Plant growth promoting bacteria and AMF not only act as biofertilizers to improve plant growth in saline soils, but also promote bioremediation of contaminated soils (Mokrani et al., 2020). Co-inoculation of different beneficial microorganisms may further improve salt tolerance (Hanin et al., 2016), which requires more attempts.

### Nanobiotechnology

Enhanced ROS scavenging improves salt tolerance in plants. Using nanoparticles (NPs) with ROS scavenging ability is an emerging approach for modulating ROS homeostasis in plants under stress conditions (Liu et al., 2021). Environmentally friendly metal-based nanomaterials can improve salt tolerance in tomato plants (Table 2). Under salinity conditions, foliar application of copper NPs (Cu-NPs) increases the content of phenolic substances in leaves and vitamin C, glutathione and phenolics in fruits, also improves the activities of phenylalanine ammonia lyase (PAL), APX, GSH-Px, SOD, and CAT. Cu-NPs induce salt tolerance of tomato by stimulating the antioxidant mechanism (Pérez-Labrada et al., 2019). Another study confirmed that the application of chitosan-polyvinyl alcohol hydrogels (Cs-PVA) and Cu-NPs activates the antioxidant defense mechanisms of tomato plants and are mediated by the octadecanoid pathway of the jasmonates (Hernández-Hernández et al., 2018). In addition to antioxidant enzyme activities, zinc oxide nanoparticles (ZnO-NPs) also enhance protein content and photosynthetic properties under salt stress, improving growth performance and alleviating the adverse effects of salinity on tomato (Faizan et al., 2021). Notably, combining NPs with other strategies may be more effective in improving salt tolerance in tomato. For example, nano-silicon application combined with grafting enhances shoot and root growth, fruit yield and quality of tomato under salt stress, and increases the contents of mineral, GA3, ABA, and Pro, indicating that this method holds promise as alternative techniques for alleviating salt stress in commercial tomato cultivars (Sayed et al., 2022). Overall, nanobiotechnology has a strong potential to modulate ROS homeostasis in plants and improve salt tolerance, contributing to sustainable agriculture (Liu et al., 2021).

The above-mentioned methods for improving salt tolerance in tomato should be combined with the cultivation approaches adopted by farmers, including crop rotation, selection of sowing and planting dates and harvest times, appropriate irrigation techniques, planting density and mulching films (Giordano et al., 2021). The evaluation of the effect of improving salt tolerance needs to comprehensively consider the yield and quality of tomato under field conditions and the cost-benefit ratio (Hanin et al., 2016; Ashraf and Munns, 2022).

## Conclusion and future perspectives

A clear salt tolerance mechanism and the identification of key genes in tomato plants that respond to salt stress will contribute to the fast-track breeding of salt tolerant varieties. In this review, we described the effect of salt stress on tomato growth and development, the mechanisms of tomato response to salinity, and the methods to improve tomato salt tolerance. High salt induces osmotic stress and ionic imbalance, thus inhibiting the growth and photosynthesis of tomato plants. Coping these adverse effects tomato plants have adopted strategies including maintaining ion homeostasis, improving osmotic regulation ability and antioxidant enzyme activities. At present, several problems in the regulation mechanism of salt tolerance in plants need to be resolved, including perception of Na^+^, transport and detoxification mechanisms of Cl^–^, mapping of toxicity at cell and tissue levels, new QTLs that improve salt tolerance, and cross-effect of different hormones under salt stress (Ismail and Horie, 2017; Isayenkov and Maathuis, 2019; Zhao et al., 2020).

Tomato has a more stable genetic transformation system, that is conducive to improve its salt tolerance with the introduction of foreign genes via transformation (Cuartero et al., 2006). Although, overexpression of some genes in tomato has improved its salt tolerance, but the development of salt-tolerant varieties through genetic transformation has not been fully understood yet. The evaluation of salt tolerance in transgenic plants under laboratory or greenhouse conditions has little correlation with salt tolerance under field conditions, because the field environment is a compound stress, not just salt stress (Yamaguchi and Blumwald, 2005). Genetic transformation of the tomato transcription factors involved in salt stress may partially solve the problem as in field conditions the transgenic plants do not exhibits outstanding salt tolerance, because transcription factors are generally involved in a various stresses and initiate expression of multiple target genes (Cui et al., 2018; Waseem et al., 2019; Gao et al., 2020). Salt tolerance of tomatoes in the field can be evaluated through unmanned aerial vehicle-based phenotyping using morphometric and spectral analysis (Johansen et al., 2019).

Many genes involved in salt stress response are inducible expression type, while most transgenic plants are obtained by using constitutive promoters to drive the continuous expression of target genes, which may cause unexpected side effects. This could be alleviated by replacing constitutive promoters with stress-induced or tissue-specific promoters (Yamaguchi and Blumwald, 2005). For genetic transformation, special attention should be paid to the genetic background of the transformed plants, which may determine the effectiveness of salt-tolerant genes. For instance, tomato *ZF2* enhances salt sensitivity in transgenic *Arabidopsis*, whereas improves tomato salt tolerance (Hichri et al., 2014), whether this effect is also present in tomatoes with different genetic backgrounds remains to be further studied. Moreover, the safety of transgenic crops is widely questioned worldwide, and traditional breeding costs considerable resources and is a lengthy process (Ismail and Horie, 2017). As the genome editing complex is degraded in the recipient cells, genome editing is defined as non-genetically modified (GM), which is more acceptable to the public, and it can offer products that will be difficult to produce using traditional breeding methods (Kanchiswamy et al., 2015). Although major breakthroughs have been made in tomato genome editing, that mainly focuses on development, metabolism, biotic stresses, and abiotic stresses such as drought, chilling and herbicides (Xu et al., 2019), while studies on tomato salt stress are few and far between. Genome editing can simultaneously edit multiple salt stress-related genes in the same tomato variety, helping to improve salt tolerance.

Many wild tomato species harboring salt-tolerant alleles are excellent natural resources for improving salt tolerance of cultivated tomatoes (Pailles et al., 2020; Wang et al., 2020). Genome editing enables *de novo* domestication of wild tomato species without causing an associated drag on salt tolerance, accelerating the genetic improvement of wild tomato species (Li T. et al., 2018). Crossing of salt-tolerant wild tomato with salt-sensitive varieties produces segregating populations for studying the genetic structure of salt tolerance (Bai et al., 2018). In addition, salt-tolerant wild tomato species can be used as rootstocks for grafting susceptible but high yielding commercial cultivars (Singh et al., 2020). Overall, in future, we can use GWAS, genome, transcriptome and proteomic analysis to identify the new QTLs and improve the regulatory network of tomato salt stress, optimize the combination of cultivation techniques to improve salt tolerance. We can develop the identification methods of high-throughput phenotyping for tomato salt tolerance, strengthen the utilization of wild salt-tolerant species, adopt efficient gene editing and genetic transformation techniques to carry out precise breeding, and use MAS breeding to improve breeding efficiency, ultimately achieving the goal of producing high-yield and high-quality tomatoes under salinity conditions.

## Author contributions

MG, X-MW, Y-MG, and J-SL conceived and designed the manuscript. MG, X-SW, H-DG, and S-YB collected and analyzed the literatures. MG wrote the manuscript. AK, Y-MG, and J-SL reviewed the manuscript. All authors contributed to the article and approved the submitted version.
